# Variation in the chemical profiles of three foxglove species in the central Balkans

**DOI:** 10.3389/fpls.2023.1155297

**Published:** 2023-03-09

**Authors:** Uroš Gašić, Tijana Banjanac, Branislav Šiler, Jelena Božunović, Milica Milutinović, Neda Aničić, Slavica Dmitrović, Marijana Skorić, Jasmina Nestorović Živković, Luka Petrović, Miloš Todorović, Suzana Živković, Dragana Matekalo, Biljana Filipović, Tamara Lukić, Danijela Mišić

**Affiliations:** Department of Plant Physiology, Institute for Biological Research “Siniša Stanković” - National Institute of the Republic of Serbia, University of Belgrade, Belgrade, Serbia

**Keywords:** UHPLC-LTQ OrbiTrap MS, cardiac glycosides, steroids, phenylethanoids, flavonoids, *Digitalis grandiflora*, *D. lanata*, *D. ferruginea*

## Abstract

The aim of this study was to determine intra- and interspecies variation in the qualitative and quantitative composition of methanol-soluble metabolites in the leaves of three *Digitalis* species (*D. lanata*, *D. ferruginea*, and *D. grandiflora*) from the central Balkans. Despite the steady use of foxglove constituents for human health as valuable medicinal products, populations of the genus *Digitalis* (*Plantaginaceae*) have been poorly investigated to describe their genetic and phenetic variation. Following untargeted profiling using UHPLC-LTQ Orbitrap MS, by which we identified a total of 115 compounds, 16 compounds were quantified using the UHPLC(–)HESI–QqQ-MS/MS approach. In total, 55 steroid compounds, 15 phenylethanoid glycosides, 27 flavonoids, and 14 phenolic acid derivatives were identified across the samples with *D. lanata* and *D. ferruginea* showing a great similarity, while 15 compounds were characteristic only for *D. grandiflora*. The phytochemical composition of methanol extracts, considered here as complex phenotypes, are further examined along multiple levels of biological organization (intra- and interpopulation) and subsequently subjected to chemometric data analysis. The quantitative composition of the selected set of 16 chemomarkers belonging to the classes of cardenolides (3 compounds) and phenolics (13 compounds) pointed to considerable differences between the taxa studied. *D. grandiflora* and *D. ferruginea* were found to be richer in phenolics as compared to cardenolides, which otherwise predominate in *D. lanata* over other compounds. PCA revealed lanatoside C, deslanoside, hispidulin, and *p*-coumaric acid to be the main compounds contributing to the differences between *D. lanata* on one side and *D. grandiflora* and *D. ferruginea* on the other, while *p*-coumaric acid, hispidulin, and digoxin contribute to the diversification between *D. grandiflora* and *D. ferruginea*. However, quantitative variation in the metabolite content within species was faint with mild population diversification visible in *D. grandiflora* and particularly in *D. ferruginea.* This pointed to the highly conserved content and ratio of targeted compounds within the analyzed species, which was not severely influenced by the geographic origin or environmental conditions. The presented metabolomics approach might have, along with morphometrics and molecular genetics studies, a high information value for further elucidation of the relationships among taxa within the genus *Digitalis*.

## 1 Introduction

Both genotyping and phenotyping of a robust sample set of a species under study are essential to properly understand the variation in its populations. The variation itself is the main prerequisite for a species to “outwit” environmental pressures and to prolong its survival on the planet. In medicinal plants, assessing the genetic and phenetic variation of wild plant populations enables appropriate planning of selective breeding to ensure favorable yield. Besides crop and timber improvement programs, medicinal plants, being a great part of the modern world health care system (Fabricant and Farnsworth, 2001), are in the focus of plant breeders. In fact, around 25% of drugs commonly used worldwide is derived from plants (Mishra et al., 2013).

During the Middle Ages, foxglove (*Digitalis* spp., family *Plantaginaceae*) has been considered to have multifarious healing properties (Aronson and Aronson, 1986) and the first reports of its role as an agent in the congestive heart failure treatment dated from the year 1250 (Williams, 1861). Today, *D. purpurea* L. and, more notably, *D. lanata* Ehrh. are both widely collected from nature and field-cultivated to extract cardenolides, being among the best producers of these compounds in the plant world (Clemente et al., 2011). One of them, digoxin, is mainly produced in Europe from dried leaves of *D. lanata*, reaching up to 1.5% dry weight (Kennedy, 1978; Bown, 1995; Clemente et al., 2011). There is a vast diversity of cardenolides within the genus *Digitalis* with more than one hundred different compounds isolated, the most commercially attractive, besides digoxin, being lanatoside C, digitoxin, and acetyldigitoxin (Clemente et al., 2011; Kreis, 2017). Other compounds, such as digitanols, steroidal saponins, anthranoids, phenols, sterols, and polysaccharides, have been identified in various *Digitalis* species (see Clemente et al., 2011 and Kreis, 2017 for reviews). Today, with the development of synthetic substitute drugs for cardiac diseases, foxgloves are given “a second chance” (Kreis, 2017) as potent agents against various viruses as well as for the treatment of cystic fibrosis and several cancer types (Bertol et al., 2011).

Out of the 27 recognized species of the genus *Digitalis* (Plants of the World Online | Kew Science, 2023), 5 are found in the Balkan Peninsula: *D. lanata, D. laevigata* Waldst. & Kit.*, D. ferruginea* L.*, D. viridiflora* Lindl., and *D. grandiflora* Mill. (syn. *D. ambigua* Murray), the first three belonging to the section *Globiflorae* and the last two to expanded “*Maranthae*” (Bräuchler et al., 2004). Two more species, namely *D. ikarica* (P.H.Davis) Strid and *D. fuscescens* Waldst. & Kit., can be found in the Balkans, the former inhabiting several North Aegean and Dodecanese islands, while the latter being reported to represent a hybrid between *D. grandiflora* and *D. purpurea* (Bræmer et al., 1927). Among these, *D. lanata* is somewhat better studied in terms of genetic and phenetic diversity and by its potential to accumulate various specialized metabolites (Braga et al., 1997; Yücesan et al., 2018). Few data are available for the other species listed (Kennedy, 1978; Katanić et al., 2017; Kaska et al., 2020). Surprisingly, variation in metabolite fingerprints both between and within wild populations is pretty much neglected, having in mind that it can render starting material for selective breeding or at least for the selection of specific genotypes that would accumulate metabolite amounts appropriate for the pharmaceutical industry. Nevertheless, according to EURISCO (Kotni et al., 2023), many landraces are stored in seed collections. Therefore, phytochemical characterization of an appropriate sample set consisting of as many populations as possible and represented by a sufficient number of individuals is an evident prerequisite to perceive the extent of variation of specialized metabolites in *Digitalis* species. Since the Balkan Peninsula represents one of the two centers of the genus diversity and considering that *D. lanata*, *D. ferruginea*, and *D. grandiflora* are most commonly found throughout it, the three species were selected for the study. In a greater perception, *D. lanata* grows across the southeast Europe and Turkey (and has been introduced into North America and several central European and Asian countries), *D. ferruginea*, besides the Balkan Peninsula, inhabits the Apennine Peninsula, Asia Minor, and the Caucasian region, while *D. grandiflora* is spread throughout Europe (except for several westernmost and northernmost countries) and northwest Asia, but has been introduced into North America. Central Balkan populations of these three species contain few individuals (several dozen at maximum, pers. obs.), which implicates that they might be at risk of extinction due to the bottleneck effect. Therefore, the protection of degraded populations should be considered and the level of their genetic variation must be assessed (Nebauer et al., 2000). However, only *D. grandiflora* has been studied in this sense (Boronnikova et al., 2007). The detection of population differentiation by studying either genetic or phenetic variation (or preferably both) provides valuable information for planning conservation measures and conducting monitoring as well as for sampling germplasm for *ex situ* conservation (Nazir et al., 2008; Clemente et al., 2011).

The present study is aimed at determining, disentangling, and interpreting the chemical diversity of three *Digitalis* species growing in the Balkan Peninsula, to setup the footprint for meaningful biodiversity conservation strategies and to propose the means for sustainable utilization of bioresources. Furthermore, this study will establish the background for future elucidation of molecular aspects of chemical diversity by adopting state-of-the art omics technologies and will enable the development of alternative strategies for the production of bioactive compounds through biotechnology approaches. The specific objective of this study was to select high-resolution chemical markers to estimate both inter- and intrapopulation variability of *Digitalis* species by applying a targeted metabolomic approach.

## 2 Material and methods

### 2.1 Chemicals

All reagents and solvents used were of analytical grade. Acetonitrile, formic acid (both MS grade), and methanol (HPLC grade) were purchased from Merck (Darmstadt, Germany). Ultra-pure water (Water Purification System, New Human Power I Integrate, Human Corporation, Republic of Korea) was used to prepare standard solutions and blanks. Analytical standards of protocatechuic acid, syringic acid, *p*-hydroxybenzoic acid, 5-*O*-caffeoylquinic acid, caffeic acid, aesculetin, isoorientin, *p*-coumaric acid, quercetin 3-*O*-glucoside, naringin, luteolin, hispidulin, and isorhamnetin were purchased from Sigma Aldrich (Steinheim, Germany). Standards of deslanoside, lanatoside C, and digoxin were provided by Professor Yang Ye and Dr. Chunping Tang (Shanghai Institute of Materia Medica- SIMM, Chinese Academy of Sciences — CAS, China).

### 2.2 Plant material

**Figure 1:**
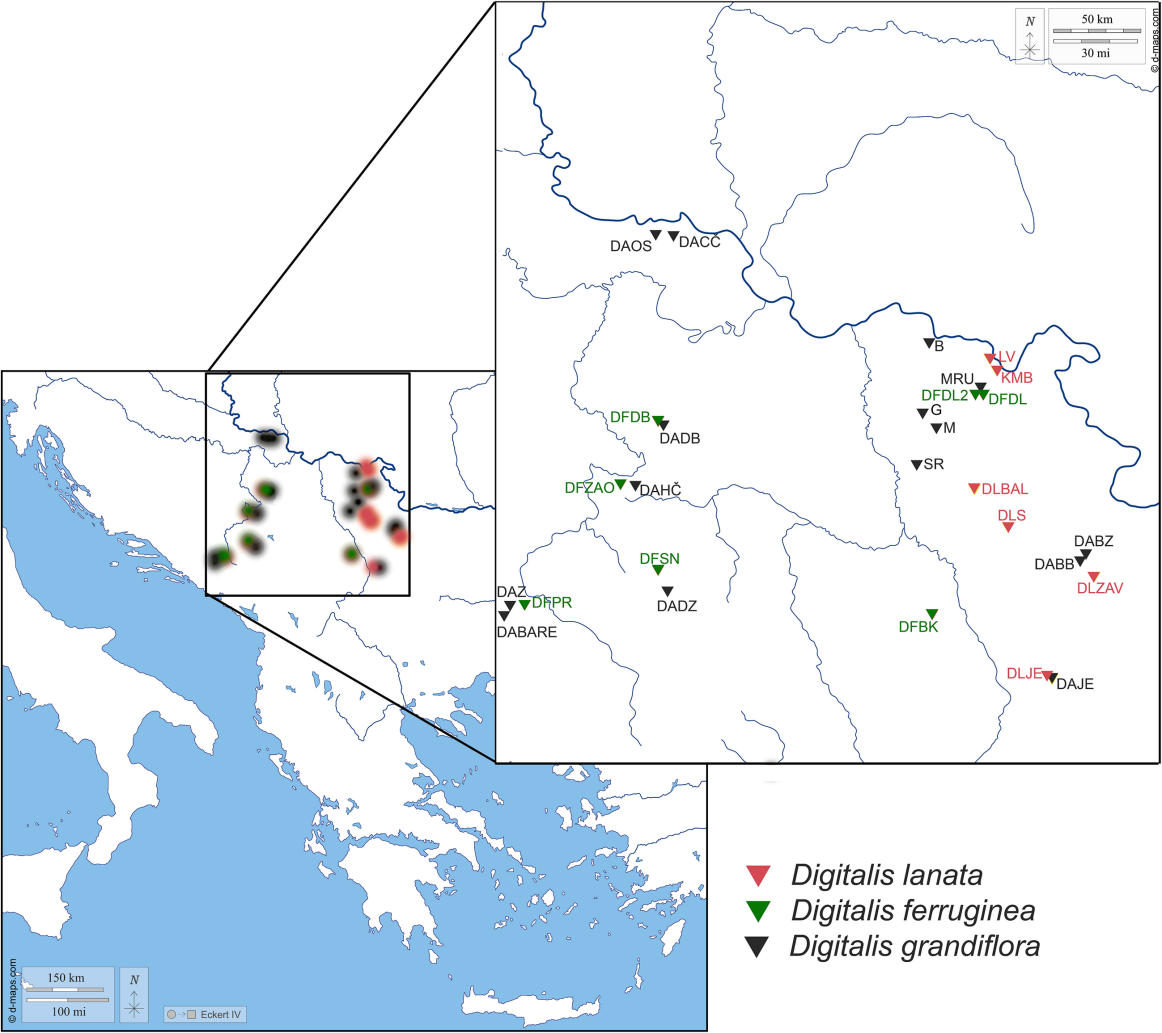
Map presenting the populations of *Digitalis grandifloraa*, *D. lanata*, and *D. ferruginea* originating from the central Balkan Peninsula analyzed within the present study. For the population labels, please refer to **Table 1**.

Leaves from the flowering stems were collected from individual plants during June, July, and August of 2020 and 2021 from wild populations in the central part of the Balkan Peninsula. Localities and the list of 28 populations of three *Digitalis* species (*Digitalis grandiflora*, *D. lanata*, and *D. ferruginea*) are listed in **Table 1** and their spatial distribution is presented in **Figure 1**. Plants were identified in the field by the authors and classified by referring to The World Flora Online (WFO) database (The World Flora Online, 2023) and Plants of the World Online | Kew Garden (Plants of the World Online | Kew Science, 2023). The corresponding voucher specimens are deposited in the herbarium of the University of Belgrade, Serbia (BEOU, acronym follows Thiers, 2023) with the voucher numbers listed in **Table 1**.

**Table 1:** The list of *Digitalis grandiflora*, *D. lanata* and *D. ferruginea* accessions used in the study.

Taxon	Population accession code (No. of studied individuals per population)*^a^*	Country, region, locality of sampled populations	Geographic latitude and longitude	Elevation [m]	Date of collection	Herbarium voucher number
***Digitalis grandiflora* Mill.**	**G** (1-10)	Serbia: Homolje Mt., Gornjak	44°15’52”N	218	June 3, 2020	17729
			21°32’40”E			
	**M** (1-7)	Serbia: Homolje Mt., Milanovac	44°12’02”N	245	June 3, 2020	17728
			21°35’50”E			
	**SR** (1-10)	Serbia: Pomoravlje, Senje	43°58’17”N	246	June 3, 2020	17726
			21°29’26”E			
	**B** (1-10)	Serbia: Đerdap National Park, Brnjica	44°39’08”N	132	June 17, 2020	17734
			21°35’59”E			
	**MRU** (1-10)	Serbia: Majdanpek, Majdanpek mine	44°24’35”N	383	June 17, 2020	17730
			21°55’21”E			
	**DABB** (1-10)	Serbia: Nature Park Stara planina, Balta Berilovac	43°24’10”N	494	June 25, 2020	17735
			22°30’43”E			
	**DABZ** (1-10)	Serbia: Nature Park Stara planina, Babin Zub	43°24’59”N	596	June 25, 2020	17727
			22°32’34”E			
	**DAJE** (1-10)	Serbia: Landscape of Outstanding Qualities “Vlasina”, Jerma	42°45’45”N	872	June 26, 2020	NA
			22°24’55”E			
	**DACČ** (1-10)	Serbia: National Park Fruška gora, Crni Čot	45° 9’22”N	440	July 30, 2020	NA
			19°49’05”E			
	**DAO**S (1-10)	Serbia: National Park Fruška gora, Orlova stena	45° 9’33”N	435	July 30, 2020	NA
			19°42’00”E			
	**DADB** (1-10)	Serbia: Jablanik Mt., Debelo brdo 1	44°10’14”N	810	August 20, 2020	17733
			19°43’10”E			
	**DAHČ** (1-10)	Serbia: National Park Tara, Hajdučka česma	43°53’22”N	1004	August 20, 2020	17731
			19°31’37”E			
	**DADZ** (1-10)	Serbia: Zlatar Mt., Drmanovići	43°25’08”N	1253	August 21, 2020	17732
			19°49’08”E			
	**DAZ** (1-7)	Bosnia and Hercegovina: Sutjeska National Park, Moštanica	43°22’46”N	726	July 20, 2021	17770
			18°39’43”E			
	**DABARE** (1-8)	Bosnia and Hercegovina: Sutjeska National Park, Donje Bare Lake	43°19’08”N	1474	July 21, 2021	17767
			18°38’18”E			
***Digitalis lanata* Ehrh.**	**LV** (1-8)	Serbia: Đerdap National Park, Lepenski Vir	44°32’51”N	110	June 17, 2020	17724
			22°01’48”E			
	**KMB** (1-10)	Serbia: Đerdap National Park, Kapetan Mišin breg	44°29’13”N	433	June 17, 2020	17725
			22°03’29”E			
	**DLBAL** (1-10)	Serbia: Rtanj Mt., Mirovo	43°48’37”N	334	June 24, 2020	NA
			21°53’10”E			
	**DLS** (1-7)	Serbia: Knjaževac, Skrobnica	43°35’25”N	585	June 24, 2020	NA
			22°05’17”E			
	**DLZAV** (1-10)	Serbia: Nature Park Stara planina, Zavojsko jezero	43°15’54”N	667	June 25, 2020	NA
			22°37’58”E			
	**DLJE** (1-7)	Serbia: Landscape of Outstanding Qualities “Vlasina”, Jerma	42°45’45”N	872	June 26, 2020	NA
			22°24’55”E			
***Digitalis ferruginea* L.**	**DFDL** (1-10)	Serbia: Majdanpečka Domena, Debeli lug 1	44°21’45”N	324	July 29, 2020	17739
			21°53’57”E			
	**DFDL2** (1-10)	Serbia: Majdanpečka Domena, Debeli lug 2	44°21’34”N	351	July 29, 2020	17738
			21°52’49”E			
	**DFDB** (1-8)	Serbia: Jablanik Mt., Debelo brdo 1	44°11’58”N	698	August 20, 2020	17740
			19°43’13”E			
	**DFZAO** (1-10)	Serbia: National Park Tara, Zaovine Lake	43°54’00”N	1097	August 20, 2020	17736
			19°25’01”E			
	**DFSN** (1-10)	Serbia: Zlatibor Mt., Seništa	43°33’06”N	1012	August 21, 2020	17737
			19°44’41”E			
	**DFPR** (1-9)	Bosnia and Hercegovina: Republika Srpska, Prijeđel	43°23’03”N	812	July 20, 2021	17768
			18°45’48”E			
	**DFBK** (1-10)	Serbia: Radan Mt., Beli Kamen	43°8’24”N	892	July 25, 2021	17771
			21°34’00”E			

^a^In total: 259 sampled individuals. NA, not available.

### 2.3 Methanol extracts preparation

After harvesting, leaves were immediately transferred into plastic zip-bags containing silica gel. Prior to methanol extraction, leaves were ground to a fine powder using liquid nitrogen. Approximately 50 mg of dry plant material was extracted with 1 ml of 80% methanol overnight at room temperature. The next day, samples were sonicated (Sonorex Bandelin Electronic, Berlin, Germany) for 1 h and subsequently centrifuged at 10,000*g* for 10 min. The supernatants were filtered through 0.2-mm cellulose filters (Agilent Technologies, Santa Clara USA) and stored at 4°C until use.

### 2.4 Identification and quantification of metabolites

#### 2.4.1 UHPLC-LTQ Orbitrap MS untargeted metabolomics analysis

Identification of metabolites in methanol extracts of the three foxglove species was done by an untargeted approach using an Accela UHPLC system connected to a linear ion trap–Orbitrap hybrid mass spectrometer (LTQ OrbiTrap XL, Thermo Fisher Scientific, Bremen, Germany) with heated electrospray ionization (HESI). Methanol extracts of *D. lanata, D. ferruginea*, and *D. grandiflora* were prepared from leaves collected in 2020 in Zavojsko jezero (population accession code No. DLZAV), Zaovine (population accession code No. DFZAO), and Debelo brdo (population accession code No. DADB), respectively (**Table 1**).

Separations of compounds were performed on a Hypersil Gold C18 column (50 × 2.1 mm, 1.9 µm; Thermo Fisher Scientific) at 40°C. The mobile phase consisted of (A) water + 0.1% formic acid and (B) acetonitrile + 0.1% formic acid. A linear gradient program at a flow rate of 0.300 mL/min was used: 0.0–1.0 min 5% (B), 1.0–14.0 min from 5% to 95% (B), 14.0–14.2 min from 95% to 5% (B), and 5% (B) for 6 min. The injection volume was 5 µL.

The mass spectrometer was operated in either negative or positive ionization mode, depending on the compound class. The HESI-source parameters were described by Koprivica et al. (2018). MS spectra were acquired by full range acquisition covering 100–1500 *m/z*. The data-dependent MS/MS events were always performed on the most intense ions detected in the full scan MS. The ions of interest were isolated in the ion trap with an isolation width of 5 ppm and activated with 35% collision energy levels.

Metabolites were identified according to the corresponding spectral characteristics: mass spectra, accurate mass, characteristic fragmentation patterns, and corresponding retention time. Tentative identification of various metabolites was achieved by studying their MS^n^ spectra and comparing them with the available literature on spectroscopic and mass data for compounds detected previously in the genus *Digitalis* and other related species **(Supplementary Table 1**). Xcalibur software (version 2.1) was used for the instrument control, data acquisition, and data analysis.

#### 2.4.2 UHPLC/(–)HESI–QqQ-MS/MS targeted metabolomics analysis

Quantification of the targeted compounds was performed using a Dionex Ultimate 3000 UHPLC system connected to a triple-quadrupole (QqQ) mass spectrometer (TSQ Quantum Access Max, Thermo Fisher Scientific, Bremen, Germany). Metabolite quantification was performed across 259 individual plants belonging to the three study species (**Table 1**).

A Syncronis C18 analytical column (100 × 2.1 mm) with 1.7 µm particle size (Thermo Fisher Scientific, Bremen, Germany) was used for the chromatographic separation. The flow rate and the composition of the mobile phases as well as the gradient elution program are described in the previous section (2.4.1.). The mass detector was equipped with a HESI source operated in the negative ionization mode. The parameters of the HESI source and the other mass detector settings were previously described by Banjanac et al. (2017).

The selected reaction monitoring (SRM) mode of the instrument was used for the quantification of the targeted compounds in the samples. The compounds were quantified by direct comparison with commercial standards. Calibration curves revealed good linearity, with *r* ^2^ values exceeding 0.99 (peak areas vs. concentration). The total amount of each compound was evaluated by calculation of the peak area and is expressed as mg/kg. The concentration of digoxin is expressed *via* the calibration curve of deslanoside, because the available amount of digoxin standard was insufficient to obtain the calibration curve with appropriate calibration levels.

### 2.5 Statistical analysis

The quantitative data were analyzed by two unsupervised methods, principal component analyses (PCA) and hierarchical cluster analyses (HCA), as well as one supervised learning method, linear discriminant analysis (LDA). For HCA the input variables were scaled to the [0, 1] range. HCA was performed based on Euclidean distances with cluster agglomeration using Ward’s (Ward, 1963) minimum variance method. LDA was performed with a presumption of the equal prior probability of classes. The correlation matrix for the quantitative data was constructed using Pearson’s correlation coefficients. All statistical analyses were performed in the Past 4 software (version 4.12; Hammer et al., 2001).

## 3 Results and discussion

Although some data on the phytochemical content of *D. grandiflora*, *D. ferruginea*, and especially *D. lanata* are available in the literature, these species are only scarcely investigated for the phytochemical interpopulation variability (Yücesan et al., 2018). Several previous studies have reported carbohydrates, iridoids, and caffeoyl phenylethanoid glycosides as suitable chemomarkers for the *Plantaginaceae* family, including the genus *Digitalis* (Taskova et al., 2005). Cardenolides and phenolics were not considered in this regard. On the other hand, tracing the amounts of commercially attractive compounds can provide valuable information to study the diversity of *Digitalis* species in the phylogenetic context. This may also highlight their characteristic populations’ metabolite profiles allowing to pick out the genotypes that produce pharmaceutically interesting compounds. More than 100 different cardenolides have been isolated from the genus *Digitali*s, and previous studies have been focused mainly on several well-known and exploited *Digitalis* species (mostly *D. purpurea* and *D. lanata*). However, to search for novel compounds, there is a necessity for in-depth screening of various *Digitalis* species for their metabolite composition, which may render bioactive agents for the treatment of various diseases. On the other hand, cardenolides occur in multi-compound mixtures whose composition represents complex phenotypes that may vary along multiple dimensions and levels of biological organization. *Digitalis* species are characterized by the presence of a vast array of phenolic compounds, among which flavonoids, mainly belonging to the flavone and 3-methoxyflavone groups, predominate. These two large groups of bioactive compounds and their diversity both within and among *Digitalis* species deserve a considerable attention. Simultaneous tracing of their composition in samples can ensure characterization of phenotypes in an ecologically and evolutionary meaningful way and enable the elucidation of the relations among and within group of compounds pointing out to the links between their biosynthetic pathways.

### 3.1 Species-specific metabolite profiles

According to Kreis (2017), HPLC-MS procedures for assessing the metabolic footprints of cardenolides found in *Digitalis* spp. are highly recommended. Identification of metabolites present in the methanol extracts of three foxglove species (*D. lanata*, *D. ferruginea*, and *D. grandiflora*) was achieved by high-resolution mass spectrometry (HRMS) in combination with MS^n^ fragmentation. The representative base peak chromatograms of the three species are shown in **Supplementary Figure 1**. Using this technique, the molecular formula of an unknown compound can be determined through the exact mass, and its structure can be proposed and solved by studying its fragmentation pathway. Using the UHPLC-LTQ OrbiTrap MS technique in both positive and negative ionization modes, totally 115 compounds were identified based on their monoisotopic masses, MS^n^ fragmentation, and previously reported MS data (**Table 2**; **Supplementary Table 1**). The consulted literature that contained NMR and MS data used to confirm the identification of the given compounds is listed in **Supplementary Table 1**. All identified compounds were classified into seven groups: steroidal glycosides (43 compounds), steroid aglycones (12 compounds), phenylethanoid glycosides (15 compounds), flavonoid glycosides (18 compounds), flavonoid aglycones (9 compounds), phenolic acid derivatives (14 compounds), and 6 compounds belonging to other classes. In addition to the expected cardiac glycosides, steroidal saponins as well as pregnane and furostanol glycosides were also present in the analyzed extracts. A detailed LC/MS qualitative analysis revealed very similar profiles of steroidal glycosides in the extracts of *D. lanata* and *D. ferruginea* leaves, in particular of digoxin, deslanoside, and lanatosides A, B, and C, which is consistent to earlier reports (Kennedy, 1978; Braga et al., 1997; Verma et al., 2014; Yücesan et al., 2018). On the other hand, these compounds were not detected in leaves of *D. grandiflora* although digoxin has been reported in small amounts in an earlier study (Kennedy, 1978). **Table 2** specifies which of the identified compounds were previously detected in any *Digitalis* species, while for a certain number of compounds it can be concluded that they were identified for the first time in *Digitalis* species.

**Table 2:** High resolution MS data on the metabolites identified in the three studied *Digitalis* species.

No	Compound name	*t*_R_, min	Molecular formula*^c^*	Calculated mass, *m/z*	Exact mass, *m/z*	Δ ppm	*D. lanata*	*D. ferruginea*	*D. grandiflora*
	*Steroidal glycosides*								
**1**	**Dihydroxyfurostan glycoside (purpureagitoside)*^b^***	6.51	C_56_H_93_ $O_{29}^{–}$	1229.58080	1229.57855	1.83	✚	✚	✚
**2**	**Furostanol glycoside 1**	6.56	C_50_H_83_ $O_{24}^{–}$	1067.52798	1067.52825	-0.25	✚	✚	✚
**3**	**Furostanol glycoside 2*^b^***	6.57	C_45_H_75_ $O_{20}^{–}$	935.48572	935.48560	0.13	–	–	✚
**4**	**Dihydroxypregnan-20-one glycoside*^b^***	6.72	C_33_H_55_ $O_{9}^{+}$	595.385157	595.384588	0.96	✚	✚	✚
**5**	**Furostanol glycoside 3**	6.81	C_50_H_83_ $O_{24}^{–}$	1067.52798	1067.52883	-0.80	✚	✚	✚
**6**	**Furostanol glycoside 4 (trigoneoside XVIIa)**	6.85	C_51_H_85_ $O_{24}^{–}$	1081.54363	1081.54329	0.31	–	–	✚
**7**	**Solanigroside**	6.88	C_62_H_103_ $O_{32}^{–}$	1359.64380	1359.64442	-0.46	–	–	✚
**8**	**Furost-20(22)-en-3,26-diol glycoside*^b^***	7.10	C_56_H_91_ $O_{28}^{–}$	1211.57024	1211.57090	-0.55	✚	✚	–
**9**	**Solanigroside J β isomer**	7.21	C_62_H_103_ $O_{32}^{–}$	1359.64380	1359.64183	1.44	✚	✚	–
**10**	**Deacetyllanatoside C (deslanoside)*^a,b^***	7.28	C_47_H_73_ $O_{19}^{–}$	941.47515	941.47296	2.33	✚	✚	–
**11**	**Trillin**	7.31	C_33_H_53_ $O_{8}^{+}$	577.374592	577.374318	0.47	✚	✚	✚
**12**	**Spirostanol glycoside 1**	7.37	C_56_H_91_ $O_{27}^{–}$	1195.57532	1195.57561	-0.24	–	–	✚
**13**	**Timosaponin A-I**	7.38	C_33_H_55_ $O_{8}^{+}$	579.390243	579.389377	1.49	✚	✚	✚
**14**	**Furostanol glycoside 5 (trigoneoside XVIIb)**	7.43	C_51_H_85_ $O_{24}^{–}$	1081.54363	1081.54367	-0.03	✚	✚	–
**15**	**Glucodigifucoside*^b^***	7.59	C_35_H_53_ $O_{13}^{–}$	681.34917	681.34991	-1.09	✚	✚	✚
**16**	**Digoxigenin glycoside (lanatoside C)*^a,b^***	7.83	C_49_H_75_ $O_{20}^{–}$	983.48572	983.48532	0.40	✚	✚	–
**17**	**Purpurea glycoside B*^b^***	7.83	C_47_H_73_ $O_{19}^{–}$	941.47515	941.47557	-0.45	✚	✚	✚
**18**	**Digoxin related compound*^b^***	7.91	C_35_H_51_ $O_{13}^{–}$	679.33352	679.33429	-1.13	✚	✚	✚
**19**	**Digoxin*^a,b^***	7.93	C_41_H_63_ $O_{14}^{–}$	779.42233	779.42295	-0.79	✚	✚	–
**20**	**Digitoxigenin 3-*O*-digitoxoside (evatromonoside)*^b^***	8.13	C_29_H_43_ $O_{7}^{–}$	503.30143	503.30136	0.14	✚	✚	✚
**21**	**Digitalin*^b^***	8.20	C_36_H_55_ $O_{14}^{–}$	711.35973	711.36076	-1.44	✚	✚	✚
**22**	**Digitoxigenin 3-*O*-deoxyhexoside (evomonoside)*^b^***	8.28	C_29_H_43_ $O_{8}^{–}$	519.29634	519.29656	-0.43	–	–	✚
**23**	**Digoxigenin 3-*O*-dideoxyhexoside*^b^***	8.29	C_35_H_53_ $O_{12}^{–}$	665.35425	665.35503	-1.17	✚	–	✚
**24**	**Spirostanol glycoside 2*^b^***	8.38	C_56_H_91_ $O_{27}^{–}$	1195.57532	1195.57556	-0.20	✚	✚	–
**25**	**Diginatigenin glycoside (lanatoside D)*^b^***	8.88	C_47_H_73_ $O_{20}^{–}$	957.47007	957.47089	-0.86	✚	✚	✚
**26**	**Kudinoside E**	9.18	C_53_H_81_ $O_{22}^{–}$	1069.52250	1069.52224	0.24	✚	✚	✚
**27**	**Gitoxigenin 3-*O*-tridigitoxoside tetraacetate*^b^***	9.27	C_49_H_71_ $O_{18}^{–}$	947.46459	947.46529	-0.74	✚	✚	–
**28**	**Gitoxigenin glycoside (lanatoside B)*^b^***	9.28	C_49_H_75_ $O_{20}^{–}$	983.48572	983.48573	-0.01	✚	✚	–
**29**	**Furostanol glycoside 6**	9.61	C_50_H_79_ $O_{23}^{–}$	1047.50176	1047.50177	-0.01	✚	✚	✚
**30**	**Spirostanol glycoside 3*^b^***	9.63	C_56_H_91_ $O_{28}^{–}$	1211.57024	1211.56536	4.03	✚	✚	✚
**31**	**Digitoxigenin glycoside (lanatoside A)*^b^***	9.75	C_49_H_75_ $O_{19}^{–}$	967.49080	967.49158	-0.80	✚	✚	–
**32**	**Spirostanol glycoside 4*^b^***	9.75	C_50_H_81_ $O_{23}^{–}$	1049.51741	1049.51527	2.05	✚	✚	✚
**33**	**Purpurea glycoside A*^b^***	9.80	C_47_H_73_ $O_{18}^{–}$	925.48024	925.48041	-0.19	✚	✚	–
**34**	**Spirostanol glycoside 5*^b^***	9.86	C_56_H_89_ $O_{27}^{–}$	1193.55967	1193.56017	-0.42	–	–	✚
**35**	**Spirostanol glycoside 6*^b^***	10.01	C_56_H_91_ $O_{27}^{–}$	1195.57532	1195.57344	1.57	–	–	✚
**36**	**Gymsylvestroside C**	10.48	C_63_H_95_ $O_{28}^{–}$	1299.60154	1299.59951	1.56	–	–	✚
**37**	**Spirostanol glycoside 7*^b^***	10.63	C_56_H_91_ $O_{27}^{–}$	1195.57532	1195.57528	0.03	✚	✚	–
**38**	**Furostanol glycoside 7*^b^***	10.77	C_50_H_81_ $O_{22}^{–}$	1033.52250	1033.52246	0.04	–	–	✚
**39**	**Furostanol glycoside 8**	11.06	C_51_H_83_ $O_{23}^{–}$	1063.53306	1063.53228	0.74	–	–	✚
**40**	**Furostanol glycoside 9*^b^***	11.25	C_50_H_81_ $O_{22}^{–}$	1033.52250	1033.52187	0.60	✚	✚	✚
**41**	**Furostanol glycoside 10*^b^***	11.37	C_51_H_83_ $O_{22}^{–}$	1047.53815	1047.53619	1.87	✚	✚	✚
**42**	**Spirostanol glycoside 8 (dongnoside)**	11.47	C_56_H_91_ $O_{26}^{–}$	1179.58041	1179.58035	0.05	✚	✚	–
**43**	**Tetrahydroxyolean glycoside (platycoside B)**	11.64	C_54_H_85_ $O_{25}^{–}$	1133.53854	1133.53845	0.08	✚	✚	–
	***Steroid aglycones***								
**44**	**Gitogenin*^b^***	6.72	C_27_H_45_ $O_{4}^{+}$	433.332334	433.331623	1.64	✚	✚	✚
**45**	**Tigogenone*^b^***	7.31	C_27_H_43_ $O_{3}^{+}$	415.321769	415.321304	1.12	✚	✚	✚
**46**	**Tigogenin*^b^***	7.38	C_27_H_45_ $O_{3}^{+}$	417.337419	417.336463	2.29	✚	✚	✚
**47**	**Digitoxigen-3-one*^b^***	7.96	C_23_H_33_ $O_{4}^{+}$	373.238433	373.237503	2.49	✚	✚	–
**48**	**Anhydro-digitoxigenin*^b^***	8.19	C_23_H_33_ $O_{3}^{+}$	357.243519	357.242043	4.13	✚	✚	✚
**49**	**Anhydro-periplogenone*^b^***	9.00	C_23_H_31_ $O_{4}^{+}$	371.222783	371.222033	2.02	✚	✚	–
**50**	**Cortexone*^b^***	9.15	C_21_H_31_ $O_{3}^{+}$	331.227869	331.226593	3.85	–	✚	✚
**51**	**Spirostan-3,15-dione*^b^***	10.23	C_27_H_41_ $O_{4}^{+}$	429.301034	429.299954	2.52	✚	✚	✚
**52**	**Trihydroxy-pregnan-20-one 1*^b^***	11.16	C_21_H_35_ $O_{4}^{+}$	351.254083	351.253128	2.72	✚	✚	✚
**53**	**Digipurpurogenin*^b^***	11.50	C_21_H_33_ $O_{4}^{+}$	349.238433	349.237698	2.10	✚	–	–
**54**	**Deoxo-purpnigenin*^b^***	11.62	C_21_H_35_ $O_{3}^{+}$	335.259169	335.258092	3.21	✚	✚	–
**55**	**Trihydroxy-pregnan-20-one 2*^b^***	12.03	C_21_H_35_ $O_{4}^{+}$	351.254083	351.252890	3.40	✚	✚	✚
	***Phenylethanoid glycosides***								
**56**	**Decaffeoyl acteoside**	3.13	C_20_H_29_ $O_{12}^{–}$	461.16645	461.16613	0.70	–	✚	–
**57**	**Decaffeoyl acteoside isomer**	3.66	C_20_H_29_ $O_{12}^{–}$	461.16645	461.16630	0.33	–	✚	✚
**58**	**Maxoside*^b^***	4.94	C_35_H_45_ $O_{21}^{–}$	801.24588	801.24538	0.62	✚	✚	✚
**59**	**Ferruginoside B*^b^***	5.06	C_20_H_29_ $O_{13}^{–}$	477.16137	477.16187	-1.07	✚	✚	✚
**60**	**Echinacoside*^b^***	5.18	C_35_H_45_ $O_{20}^{–}$	785.25097	785.25164	-0.86	✚	✚	✚
**61**	**Scroside D*^b^***	5.48	C_30_H_37_ $O_{16}^{–}$	653.20871	653.20889	-0.28	✚	✚	✚
**62**	**Purpureaside B*^b^***	5.54	C_35_H_45_ $O_{20}^{–}$	785.25097	785.25182	-1.08	✚	✚	✚
**63**	**Lugrandoside*^b^***	5.55	C_29_H_35_ $O_{16}^{–}$	639.19306	639.19342	-0.57	✚	✚	–
**64**	**Desrhamnosyl acteoside*^b^***	5.74	C_23_H_25_ $O_{11}^{–}$	477.14024	477.14158	-2.81	✚	✚	–
**65**	**Acteoside*^b^***	5.83	C_29_H_35_ $O_{15}^{–}$	623.19814	623.19880	-1.05	✚	✚	✚
**66**	**Ferruginoside A*^b^***	5.86	C_29_H_35_ $O_{16}^{–}$	639.19306	639.19387	-1.26	✚	✚	–
**67**	**Purpureaside E*^b^***	5.88	C_36_H_47_ $O_{20}^{–}$	799.26662	799.26758	-1.20	✚	✚	✚
**68**	**Digiciliside A*^b^***	6.04	C_37_H_49_ $O_{20}^{–}$	813.28227	813.28163	0.79	✚	✚	✚
**69**	**Forsythiaside*^b^***	6.10	C_29_H_35_ $O_{15}^{–}$	623.19814	623.19947	-2.12	–	–	✚
**70**	**Ferruginoside C*^b^***	6.74	C_37_H_49_ $O_{19}^{–}$	797.28735	797.28786	-0.64	✚	✚	✚
	***Flavonoid glycosides***								
**71**	**Apigenin 6,8-di-*C*-hexoside**	4.79	C_27_H_29_ $O_{15}^{–}$	593.15119	593.15212	-1.56	✚	✚	✚
**72**	**Luteolin 7-*O*-dihexuronide**	4.96	C_27_H_25_ $O_{18}^{–}$	637.10464	637.10556	-1.44	–	–	✚
**73**	**Luteolin 7-*O*-hexosyl-hexuronide*^b^***	5.15	C_27_H_27_ $O_{17}^{–}$	623.12537	623.12695	-2.52	✚	–	✚
**74**	**6-Hydroxyluteolin 7-*O*-hexuronide*^b^***	5.18	C_21_H_17_ $O_{13}^{–}$	477.06746	477.06803	-1.19	✚	✚	✚
**75**	**6-Hydroxyluteolin 7-*O*-hexoside**	5.24	C_21_H_19_ $O_{12}^{–}$	463.08820	463.08892	-1.55	✚	✚	✚
**76**	**Apigenin 7-*O*-dihexuronide (clerodendrin)**	5.47	C_27_H_25_ $O_{17}^{–}$	621.10972	621.11100	-2.05	–	–	✚
**77**	**Chrysoeriol 7-*O*-dihexuronide**	5.59	C_28_H_27_ $O_{18}^{–}$	651.12029	651.12067	-0.59	–	–	✚
**78**	**Luteolin 7-*O*-hexoside*^b^***	5.77	C_21_H_19_ $O_{11}^{–}$	447.09329	447.09417	-1.97	✚	✚	✚
**79**	**Luteolin 7-*O*-hexuronide*^b^***	5.78	C_21_H_17_ $O_{12}^{–}$	461.07255	461.07320	-1.41	✚	✚	✚
**80**	**Nepetin 7-*O*-hexuronide*^b^***	5.94	C_22_H_19_ $O_{13}^{–}$	491.08311	491.08375	-1.30	✚	✚	✚
**81**	**Luteolin 7-*O*-hexuronide isomer*^b^***	6.06	C_21_H_17_ $O_{12}^{–}$	461.07255	461.07349	-2.04	✚	✚	–
**82**	**Apigenin 7-*O*-hexuronide**	6.27	C_21_H_17_ $O_{11}^{–}$	445.07764	445.07849	-1.93	✚	✚	✚
**83**	**6-Hydroxyluteolin 7-*O*-(6’-coumaroyl)-hexoside**	6.39	C_30_H_25_ $O_{14}^{–}$	609.12498	609.12570	-1.18	–	✚	–
**84**	**Chrysoeriol 7-*O*-hexuronide**	6.40	C_22_H_19_ $O_{12}^{–}$	475.08820	475.08879	-1.25	✚	✚	✚
**85**	**Hispidulin 7-*O*-hexuronide**	6.49	C_22_H_21_ $O_{12}^{+}$	477.10385	477.10344	0.86	✚	✚	✚
**86**	**Jaceosidin 7-*O*-hexuronide**	6.57	C_23_H_21_ $O_{13}^{–}$	505.09877	505.09916	-0.79	✚	✚	–
**87**	**Pectolinarigenin 7-*O*-hexuronide**	7.65	C_23_H_21_ $O_{12}^{–}$	489.10385	489.10464	-1.61	✚	✚	✚
**88**	**Pectolinaringenin 7-*O*-malonylhexoside**	7.99	C_26_H_27_ $O_{14}^{+}$	563.14063	563.14043	0.35	✚	✚	–
	***Flavonoid aglycones***								
**89**	**6-Hydroxyluteolin*^b^***	5.31	C_15_H_11_ $O_{7}^{+}$	303.05103	303.05014	2.92	✚	✚	✚
**90**	**Scutellarein*^b^***	5.75	C_15_H_11_ $O_{6}^{+}$	287.05611	287.05540	2.48	✚	✚	✚
**91**	**Nepetin*^b^***	6.01	C_16_H_13_ $O_{7}^{+}$	317.06668	317.06612	1.75	✚	✚	✚
**92**	**Hispidulin*^a,b^***	6.49	C_16_H_13_ $O_{6}^{+}$	301.07176	301.07111	2.16	✚	✚	✚
**93**	**Luteolin*^a,b^***	7.22	C_15_H_9_ $O_{6}^{–}$	285.04046	285.04091	-1.57	✚	✚	✚
**94**	**Apigenin*^a,b^***	7.90	C_15_H_9_ $O_{5}^{–}$	269.04555	269.04590	-1.31	✚	✚	✚
**95**	**Chrysoeriol*^b^***	8.07	C_16_H_11_ $O_{6}^{–}$	299.05611	299.05655	-1.47	✚	✚	✚
**96**	**Santin*^b^***	9.20	C_18_H_15_ $O_{7}^{–}$	343.08233	343.08289	-1.65	✚	–	–
**97**	**Pectolinaringenin*^b^***	9.74	C_17_H_15_ $O_{6}^{+}$	315.08741	315.08632	3.46	✚	✚	✚
	***Phenolic acid derivatives***								
**98**	**Dihydroxybenzoic acid hexoside**	1.65	C_13_H_15_ $O_{9}^{–}$	315.07216	315.07223	-0.24	✚	✚	✚
**99**	**Caffeic acid hexoside**	2.30	C_15_H_17_ $O_{9}^{–}$	341.08781	341.08786	-0.14	–	–	✚
**100**	**Dihydroxybenzoic acid hexosyl pentoside**	2.31	C_18_H_23_ $O_{13}^{–}$	447.11442	447.11473	-0.70	✚	✚	✚
**101**	**Caffeic acid hexoside isomer**	2.74	C_15_H_17_ $O_{9}^{–}$	341.08781	341.08763	0.53	✚	✚	✚
**102**	**Protocatechuic acid*^a^***	3.24	C_7_H_5_ $O_{4}^{–}$	153.01933	153.01939	-0.35	–	✚	–
**103**	**Coumaric acid hexoside*^b^***	3.94	C_15_H_17_ $O_{8}^{–}$	325.09289	325.09282	0.20	✚	✚	✚
**104**	***p*-Coumaric acid*^a,b^***	4.09	C_9_H_7_ $O_{3}^{–}$	163.04007	163.04021	-0.86	✚	✚	–
**105**	**Caffeic acid*^a,b^***	4.16	C_9_H_7_ $O_{4}^{–}$	179.03498	179.03517	-1.07	✚	✚	✚
**106**	**Ferulic acid hexoside*^b^***	4.53	C_16_H_19_ $O_{9}^{–}$	355.10346	355.10375	-0.82	✚	✚	✚
**107**	**Sinapic acid hexoside**	4.62	C_17_H_21_ $O_{10}^{–}$	385.11402	385.11433	-0.81	✚	✚	✚
**108**	**Ferulic acid*^b^***	4.64	C_10_H_9_ $O_{4}^{–}$	193.05063	193.05085	-1.13	✚	✚	✚
**109**	**Sinapic acid**	4.65	C_11_H_11_ $O_{5}^{–}$	223.06120	223.06142	-0.98	✚	✚	✚
	***Other compounds***								
**110**	**Aesculin*^a^***	2.90	C_15_H_15_ $O_{9}^{–}$	339.07216	339.07202	0.40	✚	✚	✚
**111**	**Hebitol II**	3.32	C_21_H_29_ $O_{14}^{–}$	505.15628	505.15660	-0.64	✚	–	–
**112**	**Aesculetin*^a^***	4.07	C_9_H_5_ $O_{4}^{–}$	177.01933	177.01950	-0.95	✚	✚	✚
**113**	**Syringaresinol**	6.20	C_22_H_25_ $O_{8}^{–}$	417.15549	417.15671	-2.91	✚	✚	✚
**114**	**Digitoemodin*^b^***	8.56	C_15_H_9_ $O_{4}^{–}$	253.05063	253.05134	-2.80	✚	✚	✚
**115**	**ω-Hydroxyziganein-1-methyl ether*^b^***	11.13	C_16_H_11_ $O_{5}^{–}$	283.06120	283.06149	-1.05	✚	✚	✚

^a^Confirmed using available standards; ^b^Previously identified in *Digitalis* species; ^c^Molecular formula indicated which compounds were identified in the negative and which in the positive ionization mode; t_R_ – retention time (min); Δ ppm – mean mass accuracy. ✚ stands for detected and – for not detected compound.

The largest number of identified compounds (55 in total) belongs to the group of steroids (cardenolides, pregnane glycosides, furostane-type and spirostane-type steroidal saponins), 43 of which are steroidal glycosides and 12 are steroidal aglycones. Different sugar derivatives can be found as sugar components of these cardenolides and other steroid glycosides. The most common is digitoxose, which gives 130 Da as a neutral loss in the mass spectrum, followed by different hexoses (162 Da), acetyldigitoxose (172 Da), digitalose (160 Da), and less often deoxyhexose (146 Da) (Ravi et al., 2020). As can be seen from **Table 2**, some of these steroid derivatives are named after a certain *Digitalis* species (e.g., digitalin, purpureagitoside, lanatoside, purpurea glycoside), while some compounds are named trivially as furostanol or spirostanol glycosides. All derivatives of steroidal aglycones are, due to their polarity, identified only in the positive ionization mode. For all listed compounds (except compound 23) from the group of steroids, **Supplementary Table 1** lists the literature sources in which the given compounds were previously isolated or identified. Compound 23 was identified as digoxigenin 3-*O*-dideoxyhexoside and its proposed structure and fragmentation pathway are shown in **Supplementary Figure 2**. The MS^2^ base peak of this compound at 519 *m/z* resulted from the loss of one deoxyhexose residue (146 Da), while the loss of one more residue of deoxyhexose (146 Da) yields the mass of the deprotonated aglycone digoxigenin at 373 *m/z* (MS^3^ base peak). Further, neutral loss of H_2_O (18 Da) and CO_2_ (44 Da) results in MS^4^ base peak at 311 *m/z*. Interestingly, a compound with this structure has not yet been identified but has been synthesized as a digitoxin-related compound and used to test its anticytomegalovirus activity (Cai et al., 2014).

A total of 15 compounds was identified from the group of phenylethanoid glycosides (PG), all of which except decaffeoyl acteoside isomers (compounds 56 and 57) were previously identified in various *Digitalis* species (**Table 2**). All the three examined *Digitalis* species proved to be rich in PG, as previously reported (Brieger et al., 1995), except for compound 69 (forsythiaside), which was identified only in the extract of *D. grandiflora*. Compounds from the group of phenylethanoid glycosides specific for *Digitalis* have been shown to be potential tumor inhibitors - cytotoxic activity of the PG isolated from *D. davisiana* showed a strong impact on HEp-2 cells (Kutluay et al., 2019), while PG extracts from *D. purpurea* showed PKCα-inhibitory bioactivity (Zhou et al., 1998).

All 18 identified flavonoid glycosides belong to the flavone subgroup and are represented by hydroxyl, methyl, and methoxy derivatives of apigenin and luteolin (**Table 2**). Compound 71 (apigenin 6,8-di-*C*-hexoside) is a *C*-glycoside, while all others (compounds 72-88) are identified as 7-*O*-glycosides. Flavonoid *C*-glycosides were not found to be common for *Digitalis* species, but compound 71 was previously identified in the related genus *Plantago*, belonging to the same family *Plantaginaceae* (Kawashty et al., 1994). Among the derivatives of flavonoid *O*-glycosides, hexosides (neutral loss of 162 Da), hexuronides (neutral loss of 176 Da), and two acylhexosides (compound 83 and 88) were found. The presence of three glycosides (compounds 73, 76, and 77) with two hexuronic acid units was confirmed by the specific MS^2^ fragmentation, in which the fragment 351 *m/z* (dihexuronyl–H) was formed as a base peak. Usually, the MS^2^ base peak actually represents the mass of the deprotonated aglycone, which was not the case here, but the disaccharide residue had the highest abundance. In the next fragmentation stage, the MS^3^ base peak at 193 *m/z* was formed by the loss of 158 Da (dihexuronyl–H_2_O). A similar fragmentation pathway was noticed for compound 73 (luteolin 7-*O*-hexosyl-hexuronide), with the difference that its MS^2^ base peak corresponds to the mass of the deprotonated aglycone – luteolin (285 *m/z*). It was previously reported that leaves of *D. purpurea* contained luteolin 7-*O*-hexosyl-hexuronide (Harborne, 1963). Compound 86 (jaceosidin 7-*O*-hexuronide) has not been detected in *Digitalis* species so far, but its aglycone jaceosidin (Imre et al., 1984) and 7-*O*-glucoside of jaceosidin (Hiermann et al., 1977) have been reported. Using the exact mass of compound 88 with molecular ion at 563.14063 *m/z* in the positive ionization mode, its molecular formula C_26_H_26_O_14_ was generated. By studying its fragmentation pattern, it was concluded that dihydroxy-dimethoxyflavone, malonic acid and hexose had to be present in its structure. In the first stage of fragmentation, the malonylhexose unit was lost and the MS^2^ base peak at 315 *m/z* was formed, which corresponds to the mass of protonated dihydroxy-dimethoxyflavone. Further, in MS^3^ fragmentation, the loss of the CH_3_ group (15 Da) resulted in only one fragment at 300 *m/z*, while in MS^4^ fragmentation, a base peak at 168 *m/z* was observed. The fragment ion at 168 *m/z* was formed by the specific RDA fragmentation of flavonoids and it can be designated as a ^1,3^A^+^–CH_3_ fragment (Tsimogiannis et al., 2007), which further leads to the conclusion that this flavonoid has two hydroxyl groups and one methoxy group on the A ring. In view of all the above-mentioned facts, 6-methoxy-4’-methylapigenin or pectolinaringenin was proposed as the aglycone of this compound, which was otherwise common for *Digitalis* species (Imre et al., 1984). A proposed structure and a fragmentation pathway of this compound, trivially assigned as pectolinaringenin 7-*O*-malonylhexoside, is depicted in **Supplementary Figure 3**.

Among the nine identified flavonoid aglycones (**Table 2**), eight were flavone derivatives and only one (compound 96) was identified as flavonol – santin. Santin belongs to the subgroup of flavonoids termed *O*-methylated flavonols and it was earlier isolated from *D. orientalis* (Imre et al., 1984). The presence of compounds 92–94 was confirmed by comparing their mass spectra with the analytical standards, while the other compounds from this group were proven by determination of the exact masses and fragmentation pathways (ApSimon et al., 1963; Hiermann et al., 1977; Imre et al., 1984).

Derivatives of phenolic acids found in this study (**Table 2**) were previously reported to be specific for *Digitalis* species and were actually derivatives of hydroxycinnamic acid (Kırmızıbekmez et al., 2009; Katanić et al., 2017; Kaska et al., 2020). In this study, a certain number of hydroxybenzoic acid derivatives (compounds 98, 100, and 102) were also found. Three compounds (102, 104, and 105) were identified by direct comparison with the corresponding standards. A significant number of hexosyl derivatives of phenolic acids were detected, giving a specific fragmentation with the neutral loss of 162 Da.

Six compounds that structurally do not belong to any of the above-mentioned groups are included in the “other compounds” category (**Table 2**). The presence of two derivatives of coumarins, aesculetin (compound 110), and its 6-*O*-glucoside – aesculin (compound 112) was confirmed by comparison with the analytical standards. Compound 111, hebitol II (*O*-acyl carbohydrate), was found only in the *D. lanata* extract in this study, and its fragmentation was consistent with previously published MS data (Friščić et al., 2016). Compound 113, lignan syringaresinol, has not been previously detected in *Digitalis* species, but its fragmentation is in agreement with the literature data (Jiang et al., 2019). The last two compounds (114 and 115), digitoemodin and ω-hydroxyziganein-1-methyl ether, are anthraquinones previously isolated from *D. cariensis* (Imre et al., 1994).

### 3.2 Quantitative patterns of metabolites found in three *Digitalis* species

Targeted metabolic profiling was conducted with the aim of quantifying 16 bioactive compounds in methanol extracts of the three analyzed *Digitalis* species. We were able to quantify 3 compounds from the class of cardenolides: digoxin, lanatoside C, and deslanoside (syn. desacetyllanatoside C). In addition, we quantified 7 phenolic compounds, derivatives of either hydroxycinnamic (5-*O*-caffeoylquinic acid, caffeic acid, *p*-coumaric acid, and aesculetin) or hydroxybenzoic acid (*p*-hydroxybenzoic acid, syringic acid, and protocatechuic acid). Three *Digitali*s species have also been investigated with respect to their flavonoid content by targeting totally 6 compounds belonging to flavones (luteolin, hispidulin, isoorientin), flavonols (quercetin, isorhamnetin), and flavanones (naringin). The results indicate that the most abundant phenolic acids of *D. grandiflora* belong to the group of hydroxybenzoic acids (protocatechuic, syringic, and *p*-hydroxybenzoic acid), while *p*-coumaric acid predominates among hydrohycinnamic acids (Gašić et al., 2023). Dominant phenolic acids in *D. lanata* and *D. ferruginea* are *p*-coumaric acid and *p*-hydroxybenzoic acid (Gašić et al., 2023), which slightly coincides with previous findings, reporting chlorogenic and caffeic acids (Katanić et al., 2017) or ferulic acid (Kaska et al., 2020) as the most abundant in *D. ferruginea*. Hispidulin and other flavonoids belonging to the flavone and 3-methoxyflavone groups are especially abundant in *Digitalis* species (Kaska et al., 2020), which is also confirmed in the present study. Naringin, luteolin, and hispidulin are the most abundant flavonoids in *D. grandiflora* (Gašić et al., 2023). In *D. lanata* hispidulin predominates, while *D. ferruginea* leaves are characterized by the prevalence of hispidulin and naringin.

The content of the main cardenolides in *D. lanata* varies with the growth stages (Freier, 1977; Braga et al., 1997) and is further influenced by the leaves’ maturity (Vogel and Luckner, 1981), growth conditions, vegetation stage, collection time, drying method, and storage conditions and duration (Balakina et al., 2005). Within the present study, the profiling of specialized metabolites has been performed using silica gel-dried leaf samples collected from blooming plants in their second year of growth, as recommended by Clemente et al. (2011) at the stage when the content of pharmaceutically interesting compounds is at the optimal level. Leaves at the same developmental stage were collected to further reduce the effect of developmental stage on the content of compounds of interest. All the three targeted cardenolides were present in *D. lanata*, with lanatoside C predominating (Gašić et al., 2023). According to the literature, *D. lanata* is known to contain primary cardenolide glycosides including lanatoside A, B, and C series (Braga et al., 1997; Pérez-Alonso et al., 2012; Yücesan et al., 2018). On the other hand, *D. grandiflora* has been reported to contain lanatoside A and B, but lanatoside C was absent (Wichtl et al., 1987). This is in accordance with the present study, since we were able to quantify digoxin in *D. grandiflora* leaves, while lanatoside C and deslanoside were not detected or were present in trace amounts (Gašić et al., 2023). Major cardenolides in leaves of *D. ferruginea* were digoxin and deslanoside, which coincides to a previous study (Verma et al., 2014) that reported digitoxigenin and digoxigenin as the dominant cardenolides in this species.

**Figure 2:**
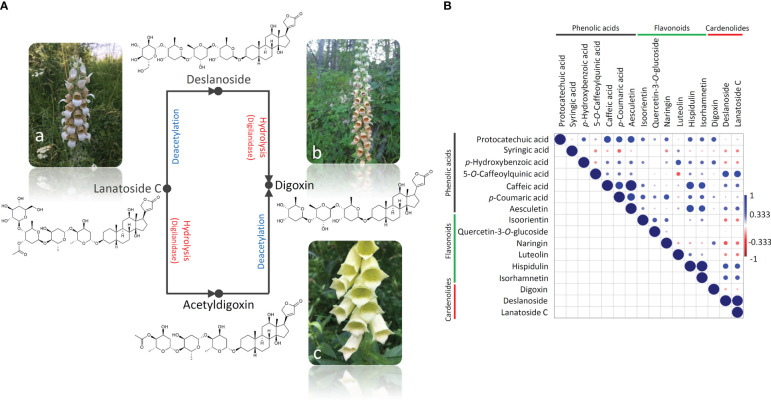
A simplified scheme of the lanatoside C hydrolysis that results in the formation of digoxin in two stages and involves the enzyme digilanidase and one deacetylation step **(A)**. The conversion goes *via* either deslanoside (desacetyllanatoside C) or acetyldigoxin. *D. lanata* (a) is a rich source of lanatoside C, *D. ferruginea* (b) contains predominantly deslanoside and digoxin, while *D. grandiflora* (c) is abundant in digoxin. Correlation analysis of the compounds’ quantitative data was performed **(B)**, and the correlation matrix was constructed using Pearson’s correlation algorithm. Color scale indicates a positive (blue) or a negative (red) correlation.

The content and the ratio of cardenolides in *Digitalis* species are regulated by the activity of enzymes responsible for the conversion of primary glycosides such as lanatoside A, lanatoside B, and lanatoside C into corresponding secondary glycosides digitoxin, gitoxin, and digoxin, respectively (Pellati et al., 2009). The hydrolysis of lanatoside C to form digoxin proceeds in two stages (**Figure 2A**) and involves the enzyme digilanidase and one deacetylation step (Cobb, 1976). Deslanoside (desacetyllanatoside C) is a product of the lanatoside C metabolism and a precursor of digoxin (**Figure 2A**). The hydrolysis of lanatoside C usually occurs after the damage of leaf tissue or plant harvesting. It has been suggested that the absence or low enzymatic activity in the plant material favors the preservation of primary glycosides in *D. lanata* (Balakina et al., 2005). On the contrary, if the enzymes are sufficiently active, a spontaneous enzymatic degradation of primary to secondary glycosides occurs, resulting in an extremely complex profile in which both primary and secondary glycosides are present in different ratios, depending on both genetic background and post-harvest processing methodologies (Balakina et al., 2005). Within the present study, harvested leaves were immediately stored on silica gel, which enabled their fast dehydration and prevented degradation of primary into secondary glycosides. Thus, we were able to trace the species- and genotype-specific content and ratio of the targeted cardenolides in *D. lanata*, *D. ferruginea*, and *D. grandiflora* leaves. Accordingly, we may conclude that *D. lanata* is characterized by the predominance of lanatoside C, which is moderately converted to digoxin, most probably *via* deslanoside (**Figure 2A**), as this intermediate is present in significant amounts in leaves of this species. The alternative digoxin precursor, acetyldigoxin, was not recorded in any analyzed taxon (**Table 2**). On the other hand, *D. grandiflora* leaves most likely display prominent activity of the enzymes responsible for the conversion of primary into secondary glycosides, as digoxin is found to be the major cardenolide in this species (Gašić et al., 2023). Lanatoside C and its metabolite deslanoside were absent in the majority of the analyzed *D. grandiflora* accessions or were present in trace amounts in several samples. In *D. ferruginea*, lanatoside C is, most likely, efficiently converted into digoxin, the dominant cardenolide compound. The conversion probably takes place *via* deslanoside intermediate, as this compound is the second most abundant compound in this species.

We further performed the pairwise correlation analysis by calculating the Pearson’s correlation coefficient (**Figure 2B**) to provide further evidence for the stated presumptions. Significant positive correlations were observed between the contents of lanatoside C and deslanoside. Digitoxin was negatively correlated with the other two cardenolides analyzed here, which is not surprising taking into account that this secondary glycoside emerges by the deacetylation of lanatoside C to deslanoside, which then undergoes hydrolysis. Cardenolides were further positively correlated with hydroxybenzoic acids and flavones hispidulin and isorhamnetin. Positive correlations were mainly observed within the group of phenolics, with the exception of syringic acid, which was negatively correlated with the majority of analyzed compounds.

**Figure 3:**
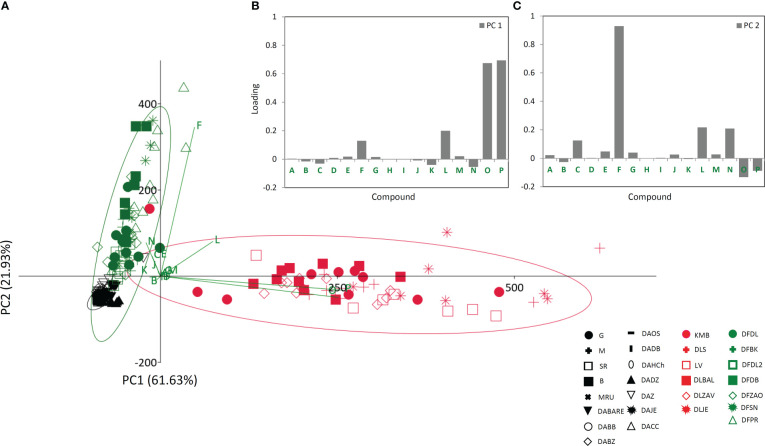
Principal component analysis (PCA) biplot with the first two PCs explaining 83.56% of total variance **(A)**. For each species 95% confidence ellipses are presented: black − *Digitalis grandiflor*a, red − *D. lanata*, green − *D. ferruginea*. Different symbols indicate affiliation of populations. Participation of the variables in the first two PCs is indicated by the corresponding vectors and by loading plots presented separately for PC1 **(B)** and PC2 **(C)**. Variables protocatechuic acid , A; syringic acid, B; *p*-hydroxybenzoic acid, C; 5-*O*-caffeoylquinic acid, D; caffeic acid, E; *p*-coumaric acid, F; aesculetin, G; isoorientin, H; quercetin-3-*O*-glucoside, I; naringin, J; luteolin, K; hispidulin, L; isorhamnetin, M; digoxin, N; deslanoside, O; lanatoside C, P. For the interpretation of population abbreviations in the figure legend, please refer to **Table 1**.

To better understand the relationship between the analyzed taxa (*D. grandiflora*, *D. lanata*, and *D. ferruginea*), we performed PCA analysis on phytochemical dataset acquired for 16 targeted compounds (**Figure 3A**). PC1 and PC2 cumulatively explained 83.56% of the total variance. *D. lanata* is clearly separated from the other two *Digitalis* species along the PC1, explaining 61.63% of the total variation. On the other hand, *D. grandiflora* and *D. ferruginea* segregate along the PC2, which explains 21.93% of the variability. The major contributors to PC1 are lanatoside C, deslanoside, hispidulin, and *p*-coumaric acid (**Figure 3B**). Along the PC2, samples are distinguished mainly by *p*-coumaric acid, hispidulin, and digoxin (**Figure 3C**). This unsupervised multivariate method offered a glimpse of possible usefulness of the selected combination of metabolites for phytochemical determination of the three *Digitalis* species. Although we presumed that *D. lanata* and *D. ferruginea*, phylogenetically closely related taxa (Bräuchler et al., 2004) would show greater similarity in phytochemical profiles, this was not the case.

Based on the HCA analysis (**Supplementary Figure S4**), a similar conclusion to that derived from PCA can be drawn regarding the phytochemical relationships among the analyzed *Digitalis* taxa. However, HCA more clearly depictured the populations’ linkages and indicated clustering of populations based on their geographical distribution.

### 3.3 Intraspecies quantitative metabolite patterns

**Figure 4:**
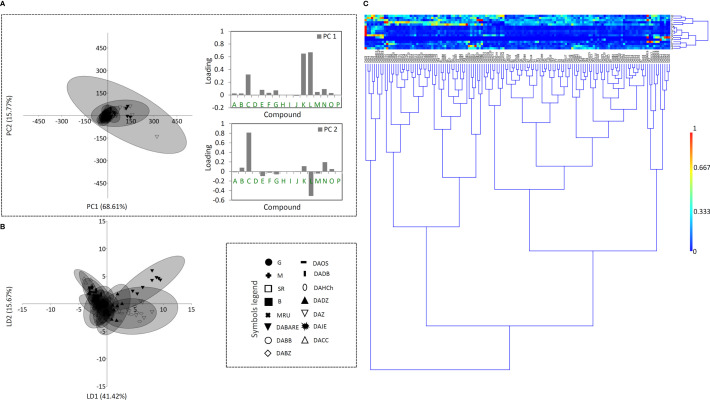
Principal component analysis (PCA) biplot with the first two PCs explaining 84.38% of the total variance **(A)** among *D. grandiflora* accessions. For fifteen *D. grandiflora* populations (each labeled by a different symbol) 95% confidence ellipses are presented. Participation of the variables (compounds) in the first two PCs is indicated by the corresponding loading plots. Linear discriminant analysis (LDA) **(B)** represents the same fifteen populations of *D. grandiflora* with 95% confidence ellipses. Heatmap of the scaled quantitative data **(C)** with the samples arranged according to the hierarchical cluster analysis (Ward’s method of cluster agglomeration). Compounds’ labels are the same as in **Figure 3**. For the interpretation of population abbreviations in the figure legend, please refer to **Table 1**.

**Figure 5:**
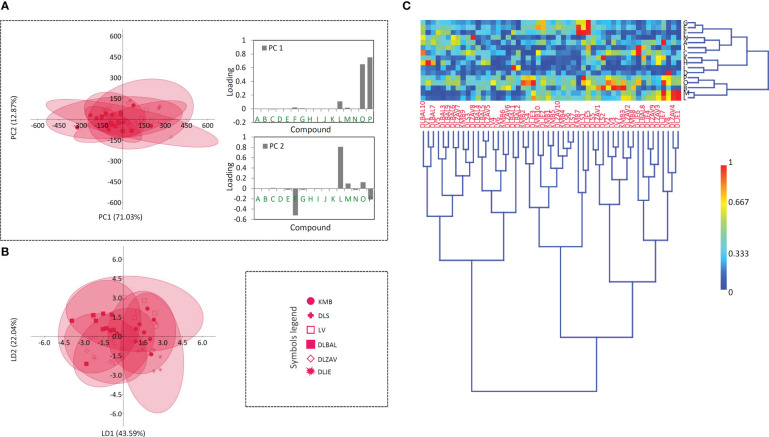
Principal component analysis (PCA) biplot with the first two PCs explaining 83.90% of the total variance **(A)** among *D. lanata* accessions. For six *D. lanata* populations (each labeled by a different symbol) 95% confidence ellipses are presented. Participation of the variables (compounds) in the first two PCs is indicated by the corresponding loading plots. Linear discriminant analysis (LDA) **(B)** represents the same six populations of *D. lanata* with 95% confidence ellipses. Heatmap of the scaled quantitative data **(C)** with the samples arranged according to the hierarchical cluster analysis (Ward’s method of cluster agglomeration). Compounds’ labels are the same as in **Figure 3**. For the interpretation of population abbreviations in the figure legend, please refer to **Table 1**.

**Figure 6:**
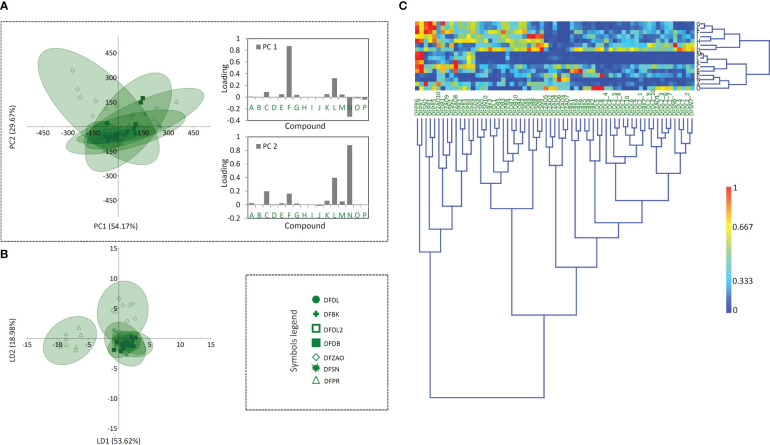
Principal component analysis (PCA) biplot with the first two PCs explaining 83.84% of the total variance **(A)** among *D. ferruginea* accessions. For seven *D. ferruginea* populations (each labeled by a different symbol) 95% confidence ellipses are presented. Participation of the variables (compounds) in the first two PCs is indicated by the corresponding loading plots. Linear discriminant analysis (LDA) **(B)** represents the same seven populations of *D. ferruginea* with 95% confidence ellipses. Heatmap of the scaled quantitative data **(C)** with the samples arranged according to the hierarchical cluster analysis (Ward’s method of cluster agglomeration). Compounds’ labels are the same as in **Figure 3**. For the interpretation of population abbreviations in the figure legend, please refer to **Table 1**.

Having in mind that distinct metabolic pathways of the 16 compounds may be reflected on different accumulation patterns across the studied populations within the three *Digitalis* species, one would expect huge quantitative variation in their profiles, since interpopulation variation in metabolite accumulation has been previously reported for other *Digitalis* species (Nebauer et al., 1999; Usai et al., 2007). However, our results imply poor population differentiation in this regard for all the three studied species. As shown in **Figures 4**–**6**, a divergence of two populations from the central sample cloud in the PCA can be observed for *D. ferruginea* (**Figure 6A**) and only mild diversification of several populations of *D. grandiflora* can be noticed in the LDA (**Figure 4B**). In particular, all individuals representing the population DABARE and 5 individuals each from DAZ and DADZ cluster separately from the main sample cloud in the HCA matrix formed by the remaining samples belonging to 12 other populations of *D. grandiflora* (**Figure 4C**). The three populations mentioned are from Dinaric karst areas with limestone as the basal rock, located at the high altitudes (**Table 1**), which could be the reason for their segregation from the common cluster, since soil characteristics are reportedly related to cardenolide production (Roca-Pérez et al., 2004). The most abundant compound in the majority of *D. grandiflora* populations, *p-*hydroxybenzoic acid, is in the same time the compound that varies at the most, followed by two flavonoid aglycones, hispidulin and luteolin (**Figure 4A**). Although present in greater amounts than in *D. lanata*, digoxin is evenly quantified across the populations of *D. grandiflora* (Gašić et al., 2023). As the species with the broadest areal among the three studied species, *D. grandiflora* shows rather surprisingly low variation in the metabolite profiles, despite being represented by the largest sample set. *D. grandiflora* is a biennial plant, occurring as a basal rosette of leaves during the first year, while the second-year rosette leaves wither rapidly during flowering stem elongation. To uniform the sampling procedure, leaves of the same developmental stage originating from flowering stems were collected for the analyses. Delayed flowering of *D. grandiflora* in higher altitudes enabled us to perform sampling throughout summer seasons and, presented results indicate that sampling period had no significant influence on the content of metabolites. An earlier study (Boronnikova et al., 2007) that estimated the partitioning of molecular variation showed that only a small fraction of the species’ genetic variation was present between populations, while most of it resided within populations (about 90%, depending on the type of molecular marker used). Therefore, the weak population differentiation seen in the metabolite profiles would most certainly have its ground in their low genetic differentiation. Further studies on the genetic background of these populations currently planned by the same team of authors will undoubtedly clarify this hypothesis.

The sample matrix made of 6 populations of *D. lanata* presented in **Figure 5A** is much more homogeneous, while clustering fails to offer the resolution to distinguish potential groups. Hispidulin and *p*-coumaric acid are the major factors of the summary metabolite variation among the studied populations of this species (**Figure 5A**). They are followed by the two most abundant cardenolide compounds in *D. lanata*, lanatoside C and deslanoside. The four compounds are also leading in the quantified amounts throughout the studied populations (Gašić et al., 2023). Nearly 50 years ago, *D. lanata* genotypes were actually quite often assessed but only with respect to cardenolide content (Weiler and Zenk, 1976; Weiler and Westekemper, 1979; Wichtl et al., 1987) and no comprehensive study based on a broader metabolite profiling has been performed in this species. However, these articles report on a remarkable variation in the accumulation of cardenolides in plants originating in different geographic regions. In our study, we were able to collect samples from 6 populations having the maximum distance of 200 km and growing in similar habitats, and only mild differences in metabolic profiles were anticipated. Sampling across a broader species’ range would most probably bring about better resolution to differentiate populations based on their metabolite footprints.

In *D. ferruginea*, the whole population DFPR along with 5 individuals from other 4 populations form a separate cluster (**Figure 6C**), while LDA differentiates the population DFZAO from the main cloud formed by the majority of the remaining individuals that belong to 5 other populations. The reason for this differentiation could be the same as for *D. grandiflora*, namely the limestone soil the plants have been grown on. The most accountable compounds for this differentiation are digoxin, hispidulin, and *p-*coumaric acid (**Figure 6A**) but for the different reasons. Hispidulin in a higher amount is quantified in the population DFPR only, while the other two compounds are the dominant specialized metabolites across the studied *D. ferruginea* populations, but their quantities vary among these (Gašić et al., 2023).

The results point to the fact that the content and ratio of targeted compounds is highly conserved within the analyzed *Digitalis* species, and is only moderately modified in response to environmental conditions, such as the soil type and altitude above sea level. To increase the resolution of discrimination at the inter- and intrapopulation level, it is possible to include the additional subset of chemomarkers, belonging to the same or other classes of metabolites, which is the course of our further work. Together with morphometrics and molecular markers, chemomarkers can provide a deeper insight into phylogenetic relationships and overall genetic variation among and within *Digitalis* taxa. Another aspect of this study provides data relevant for the conservation biology studies of the genus *Digitalis*.

The conservation of individual species, populations, and biodiversity in general is increasingly becoming one of the leading premises of applicative science. The data generated in the present study, which refer to the diversity of bioactive compounds in natural populations of three *Digitalis* species, thus representing their variation at the phenotypic level, are the starting point for the implementation of conservation strategies. As stated above, digoxin and other commercially interesting cardenolides are mainly isolated from natural sources, which might severely reduce the natural diversity in wild populations. The logical extension of this research is the study of diversity at the genotype level using various molecular markers, which is also the course of our further work.

## Data availability statement

The original contributions presented in the study are publicly available. This data can be found here: http://radar.ibiss.bg.ac.rs/handle/123456789/5448.

## Author contributions

DMi, TB, BŠ, and UG conceived and designed the experiments. TB, JB, MM, NA, SD, MS, JN, LP, MT, SŽ, DMa, BF, and TL performed the experiments. UG and DMi performed the phytochemical characterization of samples. DMi was responsible for the statistical data analysis. UG, TB, BŠ, JB, and DMi organized and wrote the manuscript with editing from all the authors. All authors contributed to the article and approved the submitted version.
