# Supplementary material for: Variation in the chemical profiles of three foxglove species in the central Balkans
Source: Front Plant Sci. 2023 Mar 9;14:1155297. doi: 10.3389/fpls.2023.1155297 (PMC10034326; doi:10.3389/fpls.2023.1155297)
Supplement: Supplementary file 1 [file DataSheet_1.docx]

Supplementary Material

Variation in the chemical profiles of three foxglove species in the central Balkans

Uroš Gašić^1*^, Tijana Banjanac^1^, Branislav Šiler^1^, Jelena Božunović^1^, Neda Aničić^1^, Milica Milutinović^1^, Slavica Dmitrović^1^, Marijana Skorić^1^, Jasmina Nestorović Živković^1^, Luka Petrović^1^, Miloš Todorović^1^, Suzana Živković^1^, Dragana Matekalo^1^, Biljana Filipović^1^, Tamara Lukić^1^, Danijela Mišić^1*^

^1^Department of Plant Physiology, Institute for Biological Research "Siniša Stanković" - National Institute of the Republic of Serbia, University of Belgrade

*** Correspondence:**Uroš Gašić
[uros.gasic@ibiss.bg.ac.rs](mailto:uros.gasic@ibiss.bg.ac.rs)

Danijela Mišić
[dmisic@ibiss.bg.ac.rs](mailto:dmisic@ibiss.bg.ac.rs)


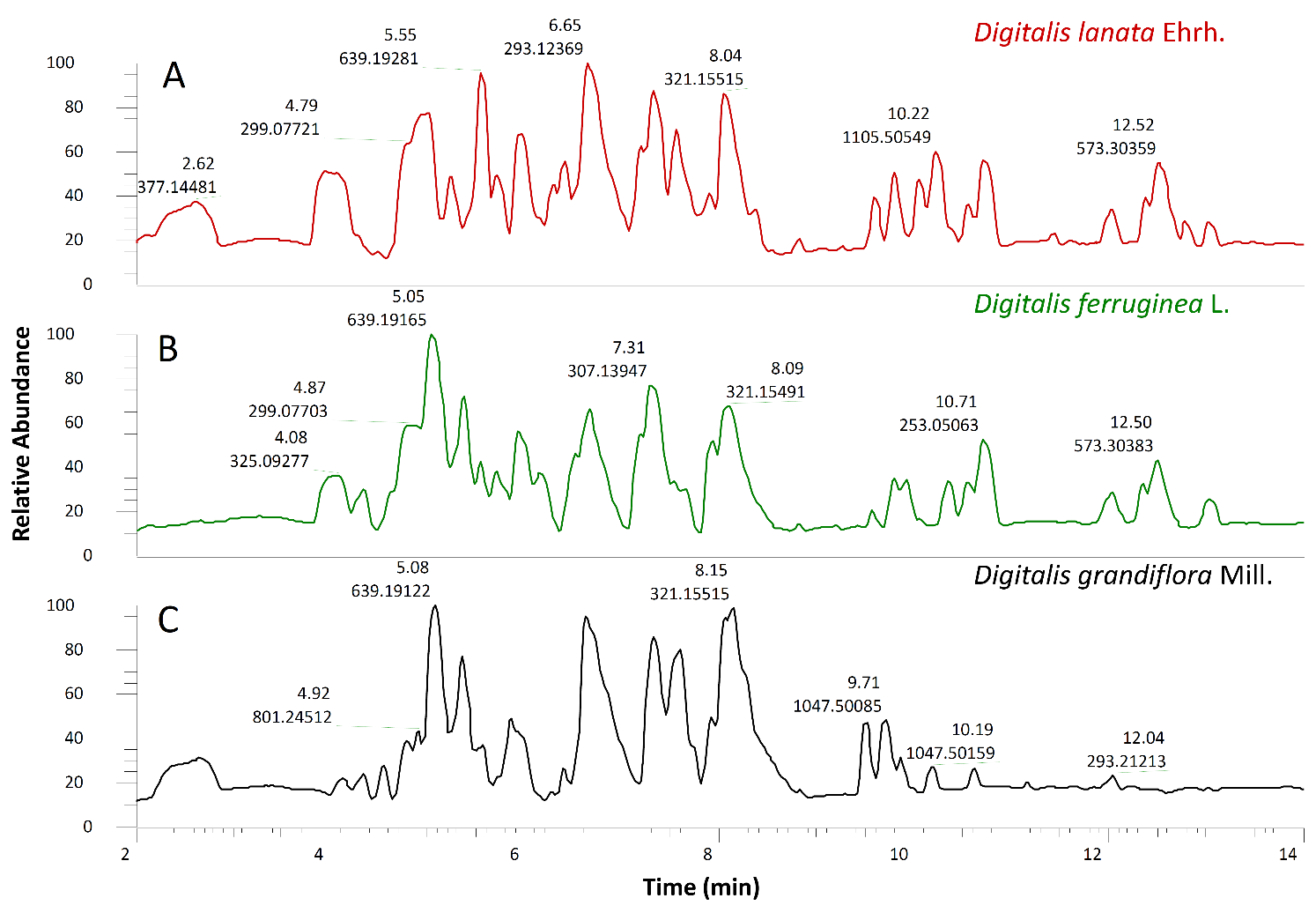


**Supplementary Figure 1**. Base peak chromatograms in negative ionization mode for all three investigated *Digitalis* species: (**A**) *D. lanata* Ehrh.; (**B**) *D. ferruginea* L. and (**C**) *D. grandiflora* Mill..





**Supplementary Figure 2**. Proposed structure and fragmentation pathway of compound **23** (digoxigenin 3-*O*-dideoxyhexoside).





**Supplementary Figure 3**. Proposed structure and fragmentation pathway of compound **88** (pectolinaringenin 7-*O*-malonylhexoside).


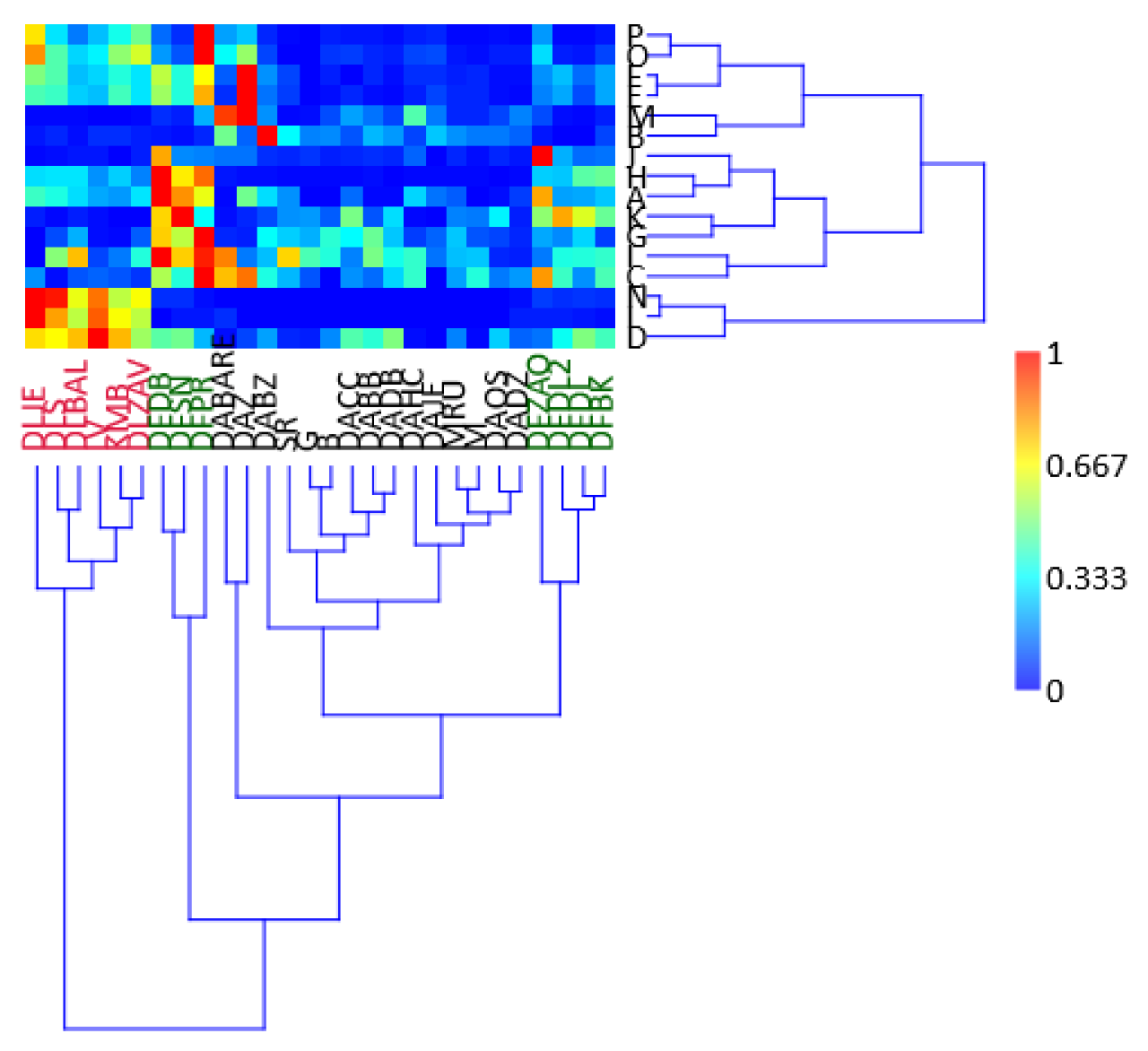


**Supplementary Figure 4**. Heatmap of the scaled quantitative data with the samples arranged according to the hierarchical cluster analysis (Ward’s method of cluster agglomeration). Mean values per population are presented for *D. grandiflora* (black letters), *D. lanata* (red letters), and *D. ferruginea* (green letters). Compounds’ labels are the same as in Figure 1. For the interpretation of population abbreviations in the figure legend, please refer to Table 1.

**Supplementary Table 1**. MS^2-4^ fragmentation data of metabolites found in three *Digitalis* species.

| **No** | **Compound name** | **Parent ion*^c^*, *m/z*** | **MS^2^ Fragments, *m/z* (% Base Peak)** | **MS^3^ Fragments, *m/z* (% Base Peak)** | **MS^4^ Fragments, *m/z* (% Base Peak)** | **References** |
| --- | --- | --- | --- | --- | --- | --- |
|  | ***Steroidal glycosides*** |  |  |  |  |  |
| **1** | **Dihydroxyfurostan glycoside (šurpureagitoside)*^b^*** | 1230 | 774(23), 936(53), 937(49), **1068**(100), 1069(95), 1098(96), 1099(86) | 774(50), **936**(100) | 612(4), **774**(100) | Skhirtladze et al., 2017 |
| **2** | **Furostanol glycoside 1** | 1068 | 774(16), 775(11), 907(4), 934(7), 935(8), **936**(100), 936(20) | 612(4), **773**(100) | 432(3), 449(32), 593(10), **611**(100), 707(4), 756(3) | da Silva et al., 2008 |
| **3** | **Furostanol glycoside 2*^b^*** | 935 | 774(52), **935**(100) | 611(5), **773**(100) | NA | Perrone et al., 2012 |
| **4** | **Dihydroxypregnan-20-one glycoside*^b^*** | 595 | 271(4), 289(48), 290(8), 431(4), **433**(100), 434(17) | 271(4), **289**(100) | 147(7), 161(43), 179(19), 201(8), 243(7), 253(96), **271**(100) | Templeton et al., 1991 |
| **5** | **Furostanol glycoside 3** | 1068 | 773(18), 775(12), 905(4), 906(4), 934(6), **936**(100), 937(30) | 612(5), **774**(100) | 449(3), 449(30), 593(13), **611**(100), 756(3) | da Silva et al., 2008 |
| **6** | **Furostanol glycoside 4 (trigoneoside XVIIa)** | 1082 | 758(15), **920**(100) | 595(7), **758**(100) | NA | Wang et al., 2017a |
| **7** | **Solanigroside** | 1360 | 904(37), 1066(35), **1198**(100), 1199(47), 1228(58), 1229(31) | 758(27), 903(83), 919(12), 1048(3), 1052(5), 1065(8), **1066**(100) | 742(3), 758(35), **904**(100), 920(22) | Wang et al., 2011 |
| **8** | **Furost-20(22)-en-3,26-diol glycoside*^b^*** | 1212 | 918(48), 919(19), 1049(15), 1050(67), 1051(30), **1080**(100), 1081(43) | 756(14), **917**(100) | **755**(100) | Skhirtladze et al., 2017 |
| **9** | **Solanigroside J β isomer** | 1360 | 904(37), 1066(35), **1198**(100), 1199(47), 1228(58), 1229(31) | 758(27), 903(83), 919(12), 1048(3), 1052(5), 1065(8), **1066**(100) | 742(3), 758(35), **904**(100), 920(22) | Wang et al., 2011 |
| **10** | **Deacetyllanatoside C (deslanoside)*^a, b^*** | 941 | 475(6), 650(9), 779(44), **780**(100) | 328(9), 345(16), **476**(100), 520(8), 606(20), 650(41), 763(17) | 328(52), 345(70), **346**(100) | Rhenius et al., 1997 |
| **11** | **Trillin** | 577 | 253(8), 271(42), 272(7), 415(12), **415**(100), 416(20), 559(3) | 253(17), **271**(100), 283(4) | 107(3), 121(7), 157(7), 159(5), 171(3), 197(4), **253**(100) | Kawasaki and Miyahara, 1963 |
| **12** | **Spirostanol glycoside 1** | 1196 | 902(15), 903(6), 918(3), **1064**(100), 1065(27) | 756(34), **901**(100), 918(25) | 740(6), **756**(100) | Pang et al., 2012 |
| **13** | **Timosaponin A-I** | 579 | 255(5), 273(49), 274(8), **417**(100), 418(17) | 255(7), **273**(100) | 145(4), 147(4), 159(5), 161(17), 163(4), **255**(100) | Kawasaki and Miyahara, 1963 |
| **14** | **Furostanol glycoside 5 (trigoneoside XVIIb)** | 1082 | 758(15), **920**(100) | 595(7), **758**(100) | NA | Wang et al., 2017a |
| **15** | **Glucodigifucoside*^b^*** | 681 | 374(8), **520**(100), 520(11) | 355(4), **373**(100) | 247(22), 309(38), **311**(100), 327(11), 329(16), 345(17), 355(8) | Ravi et al., 2020 |
| **16** | **Digoxigenin glycoside (lanatoside C)*^a, b^*** | 983 | 489(4), 762(5), 780(7), **923**(100), 925(32), 941(16), 942(8) | 475(11), 631(11), 649(25), 744(7), 761(51), **779**(100), 906(17) | 327(8), 345(18), **475**(100), 569(5), 605(12), 649(53), 761(15) | Ravi et al., 2020 |
| **17** | **Purpurea glycoside B*^b^*** | 941 | 649(3), 780(50), 781(20), 782(3), **941**(100), 943(37), 944(6) | 475(4), 649(7), **780**(100), 923(3) | 327(7), 345(21), **475**(100), 519(4), 605(14), 650(54), 761(12) | Ravi et al., 2020 |
| **18** | **Digoxin related compound*^b^*** | 679 | 345(29), **473**(100), 475(21), 517(23), 519(16), 636(40), 636(14) | 315(3), 325(11), **343**(100), 344(4), 345(20), 417(8) | 149(5), 285(12), **287**(100), 299(6), 301(5), 315(28), 325(6) | Weiler and Zenk, 1976 |
| **19** | **Digoxin*^a, b^*** | 779 | 346(13), **476**(100), 477(15), 605(18), 649(22) | 317(5), 327(19), **345**(100), 439(3), 457(16) | 301(8), 317(3), 325(12), **327**(100) | Ravi et al., 2020 |
| **20** | **Digitoxigenin 3-*O*-digitoxoside (evatromonoside)*^b^*** | 503 | 311(4), 329(4), **373**(100), 374(27) | 247(16), 309(22), **311**(100), 327(7), 329(9), 343(7), 345(13) | 119(17), 158(11), 177(25), 199(9), 283(9), 293(40), **295**(100) | Munkert et al., 2017 |
| **21** | **Digitalin*^b^*** | 711 | 504(11), **665**(100), 666(5) | **503**(100) | 311(6), 329(3), **373**(100) | Ravi et al., 2020 |
| **22** | **Digitoxigenin 3-*O*-deoxyhexoside (evomonoside)*^b^*** | 519 | 501(79), 502(4), 518(3), **519**(100), 520(5), 521(9), 547(3) | 328(15), 373(93), 457(5), 475(26), 483(6), **501**(100), 502(5) | 353(6), 369(15), 371(18), 457(10), 474(10), 483(11), **484**(100) | Ravi et al., 2020 |
| **23** | **Digoxigenin 3-*O*-dideoxyhexoside*^b^*** | 665 | 374(15), 375(4), **519**(100), 520(19) | 355(4), **373**(100) | 245(8), 247(24), 309(40), **311**(100), 327(9), 329(15), 345(10) | Cai et al., 2014 |
| **24** | **Spirostanol glycoside 2*^b^*** | 1196 | 740(15), 902(16), 1034(25), 1035(12), **1064**(100), 1065(48), 1066(16) | 740(31), **902**(100) | 577(8), **739**(100) | Pang et al., 2012 |
| **25** | **Diginatigenin glycoside (lanatoside D)*^b^*** | 957 | **799**(100), 801(25), 817(9), 859(22), 897(57), 899(19), 915(20) | 273(5), 395(35), 525(49), 655(9), **655**(100), 725(12), 751(9) | 395(10), **395**(100), 525(94) | Novković et al., 2014 |
| **26** | **Kudinoside E** | 1070 | 907(36), 936(35), **1010**(100), 1011(33), 1027(48), 1034(42) | 605(39), 619(25), **718**(100), 736(90), 848(51), 865(94), 991(51) | NA | Zhou et al., 2018 |
| **27** | **Gitoxigenin 3-*O*-tridigitoxoside tetraacetate*^b^*** | 947 | 743(6), 785(12), 786(4), **887**(100), 888(44), 906(14), 906(4) | 483(11), 613(56), 647(8), 726(11), **743**(100), 813(6), 870(5) | NA | Morita and Satoh, 1968 |
| **28** | **Gitoxigenin glycoside (lanatoside B)*^b^*** | 983 | 780(7), **924**(100), 925(21), 941(6), 941(55), 943(14), 947(5) | 475(23), 503(12), 649(25), 743(36), 761(15), **779**(100), 905(21) | **475**(100), 483(11), 485(17), 605(14), 633(13), 634(14), 649(63) | Ravi et al., 2020 |
| **29** | **Furostanol glycoside 6** | 1048 | 754(7), 755(5), 915(12), **916**(100), 917(83), 918(52), 918(14) | 607(28), **753**(100), 755(6), 769(9) | 445(5), 541(5), **607**(100) | Wang, 2020 |
| **30** | **Spirostanol glycoside 3*^b^*** | 1212 | 756(50), 1049(41), **1050**(100), 1051(71), 1080(39), 1081(28) | 609(26), 753(4), **755**(100), 757(7), 904(13), 917(87), 919(8) | 543(4), 608(3), **609**(100) | Skhirtladze et al., 2017 |
| **31** | **Digitoxigenin glycoside (lanatoside A)*^b^*** | 967 | 763(3), **907**(100), 909(37), 925(12), 926(5) | 503(21), 633(62), 745(7), **763**(100), 833(5), 860(6), 871(4) | 459(3), 503(19), **633**(100), 745(5) | Ravi et al., 2020 |
| **32** | **Spirostanol glycoside 4*^b^*** | 1050 | 756(8), 757(8), 915(68), 916(30), 917(59), **919**(100), 919(14) | 609(19), 611(34), 754(4), 755(79), **757**(100), 772(4), 773(7) | 609(25), **610**(100) | Kawasaki and Miyahara, 1965 |
| **33** | **Purpurea glycoside A*^b^*** | 925 | 503(4), 633(18), 634(6), **763**(100), 765(36), 897(7) | 503(27), 615(4), **633**(100), 746(8) | **503**(100), 615(12) | Ravi et al., 2020 |
| **34** | **Spirostanol glycoside 5*^b^*** | 1194 | 753(11), 1047(8), 1060(6), **1061**(100), 1063(30), 1064(4), 1156(4) | 542(9), 607(40), **753**(100), 915(8), 916(27), 1033(5), 1034(7) | 309(6), 372(6), 500(7), 570(6), **607**(100), 651(6), 1094(6) | Kawasaki and Miyahara, 1963 |
| **35** | **Spirostanol glycoside 6*^b^*** | 1196 | 753(11), 756(9), 1061(10), **1062**(100), 1063(32), 1064(87), 1065(43) | 445(3), 607(4), 607(49), 753(14), **753**(100), 754(4), 915(50) | 307(3), 500(7), 542(5), 589(4), **607**(100) | Pang et al., 2012 |
| **36** | **Gymsylvestroside C** | 1300 | 1212(37), 1213(19), 1230(21), 1231(13), 1258(16), **1272**(100) | 749(10), 1152(10), 1169(9), 1194(7), **1211**(100), 1230(88) | **749**(100), 1031(38), 1050(50), 1152(73), 1169(87), 1194(93) | Xu et al., 2015 |
| **37** | **Spirostanol glycoside 7*^b^*** | 1196 | 1034(3), 1149(14), **1149**(100) | 946(21), 975(7), 987(8), 1017(5), 1089(58), 1107(18), **1108**(100) | 813(25), 903(7), 934(6), **946**(100), 975(83), 1047(10), 1065(24) | Pang et al., 2012 |
| **38** | **Furostanol glycoside 7*^b^*** | 1034 | 739(7), 899(5), 900(11), 901(10), **902**(100), 903(21), 1014(4) | 593(23), 738(3), **739**(100), 756(11) | 527(6), 528(5), 582(3), **593**(100), 594(6), 788(4), 1089(3) | Kawasaki and Nishioka, 1964a |
| **39** | **Furostanol glycoside 8** | 1064 | 902(25), 903(10), 917(6), 1015(22), 1016(12), **1017**(100), 1018(5) | 754(3), 885(17), **886**(100) | 293(13), 590(8), 591(52), 682(8), 723(8), **753**(100), 754(41) | Wang et al., 2017a |
| **40** | **Furostanol glycoside 9*^b^*** | 1034 | 887(25), 888(29), 889(15), 901(29), **902**(100), 903(56), 904(38) | 448(9), 587(9), **593**(100), 738(15), 740(11), 756(65), 997(8) | NA | Kawasaki and Nishioka, 1964a |
| **41** | **Furostanol glycoside 10*^b^*** | 1048 | 724(14), 868(3), 884(4), **885**(100), 887(25), 888(3), 999(4) | 577(29), 723(13), **724**(100), 739(5) | 511(3), **577**(100) | Gvazava and Kikoladze, 2007 |
| **42** | **Spirostanol glycoside 8 (dongnoside)** | 1180 | 724(49), **1017**(100), 1019(37), 1048(40), 1131(17), 1133(55) | 577(3), 578(21), 723(5), 724(86), 872(9), 885(3), **885**(100) | 484(4), 578(18), **723**(100), 739(9) | Olvera-García et al., 2015 |
| **43** | **Tetrahydroxyolean glycoside (platycoside B)** | 1134 | 1001(14), 1048(14), 1074(37), 1075(27), 1090(30), **1092**(100) | 702(19), 797(29), **929**(100), 959(52), 960(29), 1049(16) | NA | Wang et al., 2017b |
|  | ***Steroid aglycones*** |  |  |  |  |  |
| **44** | **Gitogenin*^b^*** | 433 | 271(4), 287(7), **289**(100), 290(12), 413(10), 414(3), 415(3) | 157(7), 161(48), 179(21), 193(6), 201(9), 253(99), **271**(100) | 147(6), 159(5), 197(9), 199(5), 211(6), 243(8), **253**(100) | Gvazava and Kikoladze, 2006 |
| **45** | **Tigogenone*^b^*** | 415 | 133(29), 191(38), 209(70), 210(17), 271(59), **395**(100), 396(37) | 133(13), 165(13), 191(56), 269(10), 299(35), 359(11), **377**(100) | 213(53), 225(39), 251(74), 299(57), 317(34), **335**(100), 359(69) | Jacobs and Simpson, 1935 |
| **46** | **Tigogenin*^b^*** | 417 | 255(7), 271(4), **273**(100), 274(17), 387(3) | 145(3), 147(4), 159(5), 161(15), 163(3), **255**(100) | 145(45), 147(78), 159(97), 161(37), **173**(100), 185(42), 199(66) | Gvazava and Kikoladze, 2010 |
| **47** | **Digitoxigen-3-one*^b^*** | 373 | 201(9), 309(8), 319(6), 337(73), 338(10), **355**(100), 356(12) | 189(4), 201(9), 209(7), 291(5), 309(11), 319(8), **337**(100) | 209(15), 213(12), 277(23), 291(49), 295(13), 309(30), **319**(100) | Theurer et al., 1998 |
| **48** | **Anhydro-digitoxigenin*^b^*** | 357 | 145(13), 159(14), 161(17), 293(34), 321(21), **339**(100), 340(14) | 197(22), 213(21), 257(25), 279(48), 293(87), 303(25), **321**(100) | 211(23), 225(27), 251(15), 277(28), 279(18), **293**(100), 303(79) | Tan, 1969 |
| **49** | **Anhydro-periplogenone*^b^*** | 371 | 195(23), 289(27), 317(20), **335**(100), 336(22), 353(81), 354(19) | 195(29), 197(33), 275(47), **289**(100), 291(30), 307(35), 317(98) | 181(15), 195(17), 219(20), **233**(100), 247(48), 261(53), 274(36) | Döller and Reinhard, 1979 |
| **50** | **Cortexone*^b^*** | 331 | 213(23), 287(29), 293(17), 295(19), **311**(100), 312(27), 313(58) | 211(26), 251(31), 265(22), 267(22), 275(22), 283(19), **293**(100) | 223(17), 235(17), 249(39), 250(18), 251(47), 265(30), **275**(100) | Herl et al., 2006 |
| **51** | **Spirostan-3,15-dione*^b^*** | 429 | 285(11), 297(13), 315(17), 393(15), 409(6), **411**(100), 412(11) | 215(6), 233(6), 267(8), 285(29), 297(6), 375(18), **393**(100) | 215(9), 249(15), 267(27), 347(10), 351(12), 365(13), **375**(100) | Kawasaki and Nishioka, 1964b |
| **52** | **Trihydroxy-pregnan-20-one 1*^b^*** | 351 | 147(15), 161(18), 175(11), 219(14), 241(61), **259**(100), 260(15) | 133(16), 147(30), 159(15), 161(24), 175(14), 185(20), **241**(100) | 145(49), 159(62), 171(64), 173(37), **185**(100), 199(83), 213(34) | Smith et al., 1984 |
| **53** | **Digipurpurogenin*^b^*** | 349 | 171(14), 257(23), **275**(100), 276(13), 329(10), 330(12), 331(14) | 133(34), 145(26), 147(41), 159(41), 161(27), 173(34), **257**(100) | 145(18), 159(10), 171(7), 183(6), 215(6), **229**(100), 239(40) | DagerAlbalawi, 2016 |
| **54** | **Deoxo-purpnigenin*^b^*** | 335 | 195(41), **235**(100), 243(45), 261(45), 315(29), 317(68), 333(93) | **91**(100) | 66(53), 86(60), 87(52), 110(50), 127(52), 128(50), **159**(100) | Satoh et al., 1960 |
| **55** | **Trihydroxy-pregnan-20-one 2*^b^*** | 351 | 179(51), 241(20), 259(40), **277**(100), 289(29), 331(17), 333(29) | 135(69), 149(56), 163(40), 165(51), 179(78), 241(56), **259**(100) | 133(17), 161(31), 171(21), 175(19), 185(17), 189(15), **241**(100) | Smith et al., 1984 |
|  | ***Phenylethanoid glycosides*** |  |  |  |  |  |
| **56** | **Decaffeoyl acteoside** | 461 | 135(36), 205(20), 299(19), 315(46), 377(20), **415**(100), 417(16) | 162(32), 223(30), 235(49), 247(33), 253(70), **341**(100), 397(41) | NA | Friščić et al., 2016 |
| **57** | **Decaffeoyl acteoside isomer** | 461 | 135(46), 163(22), 205(31), **315**(100), 316(13), 415(26), 417(17) | 101(10), 119(8), 134(3), **135**(100), 143(10), 149(3), 153(3) | NA | Friščić et al., 2016 |
| **58** | **Maxoside*^b^*** | 801 | **640**(100) | 315(3), 459(18), **477**(100), 478(3) | 135(3), 143(7), 179(4), 221(6), **315**(100) | Kırmızıbekmez et al., 2009 |
| **59** | **Ferruginoside B*^b^*** | 477 | 135(12), 161(5), 179(5), 221(8), **315**(100) | 131(3), **135**(100) | NA | Calis et al., 1999a |
| **60** | **Echinacoside*^b^*** | 785 | 621(12), 622(3), 623(6), **639**(100), 640(24) | 315(4), 459(19), **477**(100) | 135(7), 161(5), 179(8), 221(12), 297(3), **315**(100) | Wang et al., 2013 |
| **61** | **Scroside D*^b^*** | 653 | 459(55), 460(11), **477**(100), 478(19), 491(3) | 135(8), 161(3), 179(4), 221(9), 297(4), **315**(100) | 101(3), 113(3), 119(3), 131(4), **135**(100), 179(3) | Jin et al., 2011 |
| **62** | **Purpureaside B*^b^*** | 785 | **623**(100), 624(14) | 443(15), **461**(100), 477(9) | 135(52), 143(9), 145(4), 153(3), 163(9), 205(35), **315**(100) | Taskova et al., 2005 |
| **63** | **Lugrandoside*^b^*** | 639 | **477**(100), 478(17), 479(4) | 135(13), 143(3), 161(15), 179(6), 297(14), **315**(100) | 113(9), 119(5), **135**(100), 143(3) | Calis et al., 1999a |
| **64** | **Desrhamnosyl acteoside*^b^*** | 477 | **161**(100), 162(7), 179(25), 297(3), 301(10), 315(14), 341(4) | **133**(100) | NA | Oh et al., 2005 |
| **65** | **Acteoside*^b^*** | 623 | 443(5), **461**(100), 462(19), 477(6), 575(7) | 135(56), 145(6), 153(5), 163(15), 205(31), **315**(100) | 101(8), 113(4), 113(9), **135**(100), 143(8), 161(4), 179(4) | Matsumoto et al., 1987 |
| **66** | **Ferruginoside A*^b^*** | 639 | 315(3), **477**(100), 478(19) | 135(13), 143(5), 161(15), 179(5), 297(11), **315**(100) | 113(8), **135**(100), 143(5), 153(3) | Calis et al., 1999a |
| **67** | **Purpureaside E*^b^*** | 799 | 605(18), 606(5), **623**(100), 624(22) | 443(14), **461**(100), 477(7) | 135(57), 143(7), 145(4), 163(14), 205(36), 297(5), **315**(100) | Jin et al., 2011 |
| **68** | **Digiciliside A*^b^*** | 813 | 473(5), 491(5), 619(53), 620(13), **637**(100), 638(26), 651(6) | 329(3), 473(51), 475(66), **491**(100) | 149(34), 161(82), 178(73), **179**(100), 221(48), 222(25), 329(61) | Skhirtladze et al., 2016 |
| **69** | **Forsythiaside*^b^*** | 623 | 351(6), 443(5), **461**(100), 462(16), 477(24), 478(7), 575(4) | 134(3), 135(57), 153(6), 163(13), 205(27), **315**(100) | 113(5), 119(9), **135**(100) | Matsumoto et al., 1987 |
| **70** | **Ferruginoside C*^b^*** | 797 | 457(3), 475(15), 476(3), 603(41), 604(9), **621**(100), 622(22) | 457(39), **475**(100) | 143(39), 145(29), 149(18), 160(21), 163(52), **205**(100), 329(28) | Calis et al., 1999b |
|  | ***Flavonoid glycosides*** |  |  |  |  |  |
| **71** | **Apigenin 6,8-di-*C*-hexoside** | 593 | 353(40), 354(9), 383(22), **473**(100), 474(20), 503(28), 575(10) | **353**(100), 383(16) | 191(3), 233(3), 282(4), 297(5), 297(45), **325**(100), 335(3) | Kawashty et al., 1994 |
| **72** | **Luteolin 7-*O*-dihexuronide** | 637 | 285(29), 286(4), **351**(100), 352(8), 381(3), 591(3), 593(9) | 113(36), 131(10), 175(31), **193**(100), 289(42), 307(8), 333(11) | 89(25), 101(24), 103(37), 113(54), **131**(100), 163(21), 175(44) | Rehecho et al., 2011 |
| **73** | **Luteolin 7-*O*-hexosyl-hexuronide*^b^*** | 623 | **285**(100), 286(8), 337(5), 357(5), 447(17), 561(5), 605(16) | 151(33), 175(97), 199(92), 217(81), **241**(100), 243(63), 257(32) | 155(13), 185(14), **197**(100), 198(82), 199(85), 213(53), 226(13) | Harborne, 1963 |
| **74** | **6-Hydroxyluteolin 7-*O*-hexuronide*^b^*** | 477 | **301**(100), 302(8), 343(5), 373(5), 397(4) | 165(62), 201(57), 211(26), 229(44), **255**(100), 257(35), 283(94) | 183(7), 199(6), 211(34), 212(13), **227**(100), 237(25), 240(40) | ApSimon et al., 1963 |
| **75** | **6-Hydroxyluteolin 7-*O*-hexoside** | 463 | **301**(100), 302(8) | 165(29), 201(34), 211(18), 229(28), 255(63), 257(23), **283**(100) | 211(4), 227(21), 237(22), 239(3), **255**(100) | Friščić et al., 2016 |
| **76** | **Apigenin 7-*O*-dihexuronide (clerodendrin)** | 621 | 269(4), **351**(100), 352(6), 487(4), 533(7), 575(26) | 113(43), 131(11), 175(34), **193**(100), 289(54), 307(7), 333(10) | 71(17), 89(36), 101(18), 103(18), 113(45), **131**(100), 175(19) | Huang et al., 2020 |
| **77** | **Chrysoeriol 7-*O*-dihexuronide** | 651 | 285(3), **351**(100), 352(10), 517(8), 633(4) | 113(39), 131(9), 175(34), **193**(100), 289(51), 307(7), 333(9) | 89(31), 101(20), 103(33), 113(81), **131**(100), 163(18), 175(79) | Wang et al., 2019 |
| **78** | **Luteolin 7-*O*-hexoside*^b^*** | 447 | 284(22), **285**(100), 286(9) | 175(73), 199(75), 213(46), 217(59), **241**(100), 243(54), 267(71) | **197**(100), 198(79), 199(48), 213(44), 213(88), 226(17), 241(18) | Hiermann et al., 1977 |
| **79** | **Luteolin 7-*O*-hexuronide*^b^*** | 461 | 145(4), 163(3), 205(3), 235(6), 265(14), **285**(100), 286(13) | 175(63), 199(53), 217(60), 239(53), 241(90), 243(48), **267**(100) | 195(4), 211(12), 223(17), **239**(100) | Hiermann, 1982 |
| **80** | **Nepetin 7-*O*-hexuronide*^b^*** | 491 | **315**(100), 316(10), 387(3) | **300**(100) | **216**(100), 227(94), 228(79), 243(68), 255(72), 271(75), 272(73) | Hiermann, 1982 |
| **81** | **Luteolin 7-*O*-hexuronide isomer*^b^*** | 461 | 175(3), 283(3), 284(3), **285**(100), 286(9), 443(4) | 175(35), 185(28), 199(35), 213(37), 239(63), 241(55), **267**(100) | 195(4), 211(8), 223(11), 225(3), **239**(100) | Hiermann, 1982 |
| **82** | **Apigenin 7-*O*-hexuronide** | 445 | 175(11), **269**(100), 270(8), 307(5) | 149(43), 151(26), 201(29), 224(28), **225**(100), 227(19), 269(22) | 169(24), 180(13), 181(98), 183(51), 196(38), **197**(100), 210(8) | Barreira et al., 2014 |
| **83** | **6-Hydroxyluteolin 7-*O*-(6'-coumaroyl)-hexoside** | 609 | **301**(100), 302(10) | 165(34), 201(45), 211(29), 229(33), 255(76), 257(29), **283**(100) | 211(5), 227(17), 237(23), 239(7), 254(6), **255**(100) | Meng et al., 2008 |
| **84** | **Chrysoeriol 7-*O*-hexuronide** | 475 | 145(3), 175(5), **299**(100), 300(14), 400(4), 801(12), 802(4) | **284**(100) | 200(25), 212(24), 227(35), 228(27), 239(22), 255(52), **256**(100) | Barreira et al., 2014 |
| **85** | **Hispidulin 7-*O*-hexuronide** | 477 | **301**(100), 302(13) | **286**(100) | 121(10), 140(6), **168**(100), 240(4), 258(21), 268(4), 285(5) | Murai et al., 2008 |
| **86** | **Jaceosidin 7-*O*-hexuronide** | 505 | **329**(100), 330(16) | **314**(100) | **299**(100) | Murai et al., 2008 |
| **87** | **Pectolinarigenin 7-*O*-hexuronide** | 489 | 175(7), **313**(100) | **298**(100) | NA | Zhang et al., 2018 |
| **88** | **Pectolinaringenin 7-*O*-malonylhexoside** | 563 | **315**(100), 316(13), 477(4) | **300**(100), 301(5) | 133(11), 135(14), **168**(100), 272(22), 285(14) | NA |
|  | ***Flavonoid aglycones*** |  |  |  |  |  |
| **89** | **6-Hydroxyluteolin*^b^*** | 303 | 163(18), 169(20), 257(24), 283(33), 284(36), 285(84), **286**(100) | 121(8), **168**(100), 256(32), 257(10), 258(27), 267(9), 268(46) | 112(19), **140**(100) | ApSimon et al., 1963 |
| **90** | **Scutellarein*^b^*** | 287 | 119(18), 123(12), 169(32), 241(31), **269**(100), 287(56), 288(14) | **119**(100), 121(22), 123(68), 151(17), 213(24), 227(27), 241(65) | 77(4), **91**(100) | Imre et al., 1984 |
| **91** | **Nepetin*^b^*** | 317 | **302**(100), 303(13), 304(4) | 137(11), 140(7), **168**(100), 169(17), 273(6), 274(94), 301(7) | 84(5), 112(16), **140**(100) | Hiermann et al., 1977 |
| **92** | **Hispidulin*^a, b^*** | 301 | **286**(100) | 121(9), 140(6), **168**(100), 169(5), 257(5), 258(28), 285(5) | 84(5), 112(17), **140**(100) | Hiermann et al., 1977 |
| **93** | **Luteolin*^a, b^*** | 285 | 151(35), 175(79), 199(72), 217(64), **241**(100), 243(47), 285(55) | 185(13), **197**(100), 198(68), 199(49), 213(46), 226(12), 241(10) | 151(10), 153(11), **169**(100), 178(23), 179(10), 180(8), 234(8) | Imre et al., 1984 |
| **94** | **Apigenin*^a, b^*** | 269 | 149(48), 151(30), 183(20), 201(28), 221(70), **225**(100), 269(33) | 169(9), 180(12), **181**(100), 182(6), 183(25), 196(15), 197(38) | 67(67), **166**(100), 229(66), 237(74), 276(66), 284(75), 338(67) | Imre et al., 1984 |
| **95** | **Chrysoeriol*^b^*** | 299 | **284**(100), 285(11) | 137(58), 200(38), 212(59), **227**(100), 255(37), 256(61) | 155(3), **183**(100), 185(3), 198(3), 199(20), 227(6) | Imre et al., 1984 |
| **96** | **Santin*^b^*** | 343 | **328**(100), 329(18) | 299(4), 312(12), **313**(100) | 269(5), 282(12), 283(9), 284(37), 285(80), 295(10), **298**(100) | Imre et al., 1984 |
| **97** | **Pectolinaringenin*^b^*** | 315 | **300**(100), 301(14) | 133(12), 135(15), **168**(100), 257(8), 272(21), 285(12), 299(8) | 84(4), 112(18), **140**(100) | Imre et al., 1984 |
|  | ***Phenolic acid derivatives*** |  |  |  |  |  |
| **98** | **Dihydroxybenzoic acid hexoside** | 315 | 97(5), 127(19), 152(14), 153(45), 157(8), **161**(100), 217(9) | **73**(100) | NA | Zengin et al., 2020 |
| **99** | **Caffeic acid hexoside** | 341 | 135(11), 161(40), 177(26), 178(5), **179**(100), 180(7), 203(10) | **135**(100) | NA | Friščić et al., 2016 |
| **100** | **Dihydroxybenzoic acid hexosyl pentoside** | 447 | 152(80), 163(60), 177(35), 207(31), 271(47), **315**(100), 401(54) | 109(11), 135(14), 152(50), **153**(100), 181(14), 225(13) | NA | Šuković et al., 2020 |
| **101** | **Caffeic acid hexoside isomer** | 341 | 135(9), 161(40), 177(38), **179**(100), 180(7), 203(8), 281(5) | **135**(100) | 82(25), 91(45), 107(100), **117**(100), 135(66), 250(30), 278(25) | Friščić et al., 2016 |
| **102** | **Protocatechuic acid*^a^*** | 153 | **109**(100), 110(16), 111(94), 113(12), 125(20), 127(21), 128(13) | 65(43), **91**(100), 127(38), 139(36), 143(37), 144(36), 221(35) | NA | Dunkić et al., 2015 |
| **103** | **Coumaric acid hexoside*^b^*** | 325 | 119(11), **145**(100), 163(81), 187(40), 191(11), 205(7), 265(14) | **117**(100) | NA | Kırmızıbekmez et al., 2014 |
| **104** | ***p*-Coumaric acid*^a, b^*** | 163 | 163(69), 187(28), 307(24), 325(71), **367**(100), 368(20), 469(52) | 117(8), 119(10), 145(97), **163**(100), 223(3), 307(12), 339(11) | **119**(100) | Katanić et al., 2017 |
| **105** | **Caffeic acid*^a, b^*** | 179 | 89(4), 133(4), 134(6), **135**(100), 136(10) | 65(31), 91(82), 92(30), 107(98), 117(69), 121(32), **135**(100) | NA | Katanić et al., 2017 |
| **106** | **Ferulic acid hexoside*^b^*** | 355 | 134(3), 175(19), **193**(100), 194(3), 217(22), 235(4) | **134**(100), 149(61), 178(37) | 91(48), **106**(100), 182(45), 217(50), 225(43), 233(46), 237(49) | Kırmızıbekmez et al., 2009 |
| **107** | **Sinapic acid hexoside** | 385 | 205(63), 206(7), **223**(100), 224(9), 247(54), 265(9), 325(6) | 149(3), **164**(100), 179(18), 208(28) | **149**(100) | Amessis-Ouchemoukh et al., 2014 |
| **108** | **Ferulic acid*^b^*** | 193 | **111**(100), 129(10), 146(13), 147(48), 148(22), 149(10), 173(17) | **67**(100), 80(11), 91(10), 92(11), 140(10), 210(10), 232(11) | NA | Katanić et al., 2017 |
| **109** | **Sinapic acid** | 223 | **164**(100), 175(20), 177(80), 179(36), 205(76), 206(31), 208(39) | **149**(100) | **121**(100) | Beara et al., 2015 |
|  | ***Other compounds*** |  |  |  |  |  |
| **110** | **Aesculin*^a^*** | 339 | 175(4), **177**(100), 178(7) | 89(4), 105(13), **133**(100), 149(6), 177(7) | **89**(100), 105(90), 108(59), 140(61), 199(66), 217(61), 235(64) | Pommerrenig et al., 2019 |
| **111** | **Hebitol II** | 505 | 161(57), 179(40), 281(20), **341**(100), 342(15), 343(15), 457(13) | 135(10), **179**(100), 251(3) | **135**(100) | Friščić et al., 2016 |
| **112** | **Aesculetin*^a^*** | 177 | 85(57), 113(60), **115**(100), 129(38), 131(52), 133(97), 157(46) | 59(50), 73(28), 79(13), 85(56), **87**(100), 150(13), 235(13) | NA | Beara et al., 2015 |
| **113** | **Syringaresinol** | 417 | 151(17), 166(42), 179(19), **181**(100), 371(16), 373(29), 402(40) | **166**(100) | **151**(100) | Jiang et al., 2019 |
| **114** | **Digitoemodin*^b^*** | 253 | 177(14), 205(17), **207**(100), 209(16), 225(40), 253(90) | 87(18), 113(13), 119(14), 151(17), 163(95), **179**(100), 207(17) | NA | Imre et al., 1994 |
| **115** | **ω-Hydroxyziganein-1-methyl ether*^b^*** | 283 | **268**(100), 269(11) | **240**(100) | NA | Imre et al., 1994 |

*^a^*Confirmed using available standards; *^b^*Previously identified in *Digitalis* species; *^c^*The parent ion is rounded to a whole number to see exactly what the neutral loss was. NA – not avalible;

**References**

Amessis-Ouchemoukh, N., Abu-Reidah, I. M., Quirantes-Piné, R., Rodríguez-Pérez, C., Madani, K., Fernández-Gutiérrez, A., et al. (2014). Tentative characterisation of iridoids, phenylethanoid glycosides and flavonoid derivatives from *Globularia alypum* L. (Globulariaceae) leaves by LC-ESI-QTOF-MS. *Phytochem. Analysis* 25, 389–398. doi: 10.1002/pca.2506.

ApSimon, J. W., Haynes, N. B., Sim, K. Y., and Whalley, W. B. (1963). 705. 5, 7, 4′-Trihydroxy-3′, 6-dimethoxyflavone, a pigment from *Digitalis lanata* L. *J. Chem. Soc.*, 3780–3782. doi: 10.1039/JR9630003780.

Barreira, J. C., Dias, M. I., Živković, J., Stojković, D., Soković, M., Santos-Buelga, C., et al. (2014). Phenolic profiling of *Veronica* spp. grown in mountain, urban and sandy soil environments. *Food Chem.* 163, 275–283. doi: 10.1016/j.foodchem.2014.04.117.

Beara, I., Živković, J., Lesjak, M., Ristić, J., Šavikin, K., Maksimović, Z., et al. (2015). Phenolic profile and anti-inflammatory activity of three *Veronica* species. *Ind. Crop. Prod.* 63, 276–280. doi: 10.1016/j.indcrop.2014.09.034.

Cai, H., Wang, H.-Y. L., Venkatadri, R., Fu, D.-X., Forman, M., Bajaj, S. O., et al. (2014). Digitoxin analogues with improved anticytomegalovirus activity. *ACS Med. Chem. Lett.* 5, 395–399. doi: 10.1021/ml400529q.

Calis, I., Tasdemir, D., Sticher, O., and Nishibe, S. (1999a). Phenylethanoid Glycosides from *Digitalis* *ferruginea* subsp. *ferruginea* (=*D. aurea* LINDLEY) (Scrophulariaceae). *Chem. Pharm. Bull.* 47, 1305–1307. doi: 10.1248/cpb.47.1305.

Calis, İ., Akbay, P., Kuruuzum, A., YALCIN, F., Sahin, P., and Pauli, G. F. (1999b). Phenylethanoid and cardioactive glycosides from *Digitalis ferruginea*. *Pharmazie* 54. doi: 10.1002/chin.200010190.

da Silva, B. P., and Parente, J. P. (2008). Three new steroidal saponins from *Smilax officinalis*. *Z. Naturforsch. PT. B* 63, 95–100. doi: 10.1515/znb-2008-0114.

DagerAlbalawi, M. A. (2016). Chemistry, spectroscopic characteristics and biological activity of natural occurring cardiac glycosides. *J. Biotechnol. Biochem.* 2, 20–35.

Döller, P. C., and Reinhard, E. (1979). Biotransformation of Cardenolides: Comparative Studies with Cell Cultures of *Thevetia neriifolia* and *Digitalis lanata*. *Planta Med.* 37, 277–288. doi: 10.1055/s-0028-1097341.

Dunkić, V., Kosalec, I., Joze Košir, I., Potočnik, T., Čerenak, A., Zovko Končić, M., et al. (2015). Antioxidant and antimicrobial properties of *Veronica spicata* L. (Plantaginaceae). *Curr. Drug. Targets* 16, 1660–1670. doi: 10.2174/1389450116666150531161820.

Friščić, M., Bucar, F., and Hazler Pilepić, K. (2016). LC-PDA-ESI-MSn analysis of phenolic and iridoid compounds from *Globularia* spp. *J. Mass Spectrom.* 51, 1211–1236. doi: 10.1002/jms.3844.

Gvazava, L. N., and Kikoladze, V. S. (2006). Steroidal glycosides from *Digitalis ciliata* leaves. *Chem. Nat. Compd.* 42, 562–566. doi: 10.1007/s10600-006-0214-3.

Gvazava, L. N., and Kikoladze, V. S. (2007). Structure of a steroid saponin from *Digitalis ciliata*. *Chem. Nat. Compd.* 43, 162–165. doi: 10.1007/s10600-007-0069-2.

Gvazava, L. N., and Kikoladze, V. S. (2010). Furostane-type steroidal saponin from *Digitalis ciliata*. *Chem. Nat. Compd.* 46, 246–249. doi: 10.1007/s10600-010-9579-4.

Harborne, J. B. (1963). Plant polyphenols. X. Flavone and aurone glycosides of *Antirrhinum*. *Phytochemistry* 2, 327–334. doi: 10.1016/S0031-9422(00)84856-6.

Herl, V., Fischer, G., Müller-Uri, F., and Kreis, W. (2006). Molecular cloning and heterologous expression of progesterone 5β-reductase from *Digitalis lanata* Ehrh. *Phytochemistry* 67, 225–231. doi: 10.1016/j.phytochem.2005.11.013.

Hiermann, A. (1982). Neue Flavonglykoside in den Blättern von *Digitalis lanata*. *Planta Med.* 45, 59–60. doi: 10.1055/s-2007-971247.

Hiermann, A., Kartnig, T., Seligmann, O., and Wagner, H. (1977). Flavonoids in the leaves of *Digitalis lanata* (Ehrhart). *Planta Med.* 32, 24–26. doi: 10.1055/s-0028-1097553.

Huang, W., Wen, Z., Wang, Q., Chen, R., Li, Z., Feng, Y., et al. (2020). The chemical profile of active fraction of *Kalimeris indica* and its quantitative analysis. *Biomed. Chromatogr.* 34, e4828. doi: 10.1002/bmc.4828.

Imre, S., Ertürk, S., and Imre, Z. (1994). Two new anthraquinones from *Digitalis cariensis*. *Z. Naturforsch. C* 49, 684–686. doi: 10.1515/znc-1994-9-1020.

Imre, S., Islimyeli, S., Öztunc, A., and Büyüktimkin, N. (1984). Flavonoid aglycones in some *Digitalis* species. *Planta Med.* 50, 360–360. doi: 10.1055/s-2007-969735.

Jacobs, W. A., and Simpson, J. C. (1935). The *Digitalis* sapogenins. *J. Biol. Chem.* 110, 429–438. doi: 10.1016/S0021-9258(18)75131-4.

Jiang, Y., Liu, R., Chen, J., Liu, M., Liu, M., Liu, B., et al. (2019). Application of multifold characteristic ion filtering combined with statistical analysis for comprehensive profiling of chemical constituents in anti-renal interstitial fibrosis I decoction by ultra-high performance liquid chromatography coupled with hybrid quadrupole-orbitrap high resolution mass spectrometry. *J. Chromatogr. A* 1600, 197–208. doi: 10.1016/j.chroma.2019.04.051.

Jin, Q., Jin, H.-G., Shin, J.-E., Hong, J.-K., and Woo, E.-R. (2011). Phenylethanoid Glycosides from *Digitalis* *purpurea* L. *B. Kor. Chem. Soc.* 32, 1721–1724. doi: 10.5012/BKCS.2011.32.5.1721.

Katanić, J., Ceylan, R., Matić, S., Boroja, T., Zengin, G., Aktumsek, A., et al. (2017). Novel perspectives on two *Digitalis* species: Phenolic profile, bioactivity, enzyme inhibition, and toxicological evaluation. *S. Afr. J. Bot.* 109, 50–57. doi: 10.1016/j.sajb.2016.12.004.

Kawasaki, T., and Miyahara, K. (1963). Thin Layer Chromatography of Steroid Saponins and their Derivatives. *Chem. Pharm. Bull.* 11, 1546–1550. doi: 10.1248/cpb.11.1546.

Kawasaki, T., and Miyahara, K. (1965). Structure of yononin: a novel type of spirostanol glycoside. *Tetrahedron* 21, 3633–3639. doi: 10.1016/S0040-4020(01)96978-9.

Kawasaki, T., and Nishioka, I. (1964a). *Digitalis* Saponins. II. Leaf Saponins of *Digitalis purpurea* L. *Chem. Pharm. Bull.* 12, 1311–1315. doi: 10.1248/cpb.12.1311

Kawasaki, T., and Nishioka, I. (1964b). Digitalis Saponins. I. Seed Saponins of *Digitalis* purpurea L.(Commercial" Digitonin"). *Chem. Pharm. Bull.* 12, 1250–1253. doi: 10.1248/cpb.12.1250.

Kawashty, S. A., Abdalla, M. F., and Saleh, N. A. M. (1994). Flavonoids of *Plantago* species in Egypt. *Biochem. Syst. Ecol.* 22, 729–733. doi: 10.1016/0305-1978(94)90058-2.

Kırmızıbekmez, H., Masullo, M., Festa, M., Capasso, A., and Piacente, S. (2014). Steroidal glycosides with antiproliferative activities from *Digitalis trojana*. *Phytother. Res.* 28, 534–538. doi: 10.1002/ptr.5012.

Kırmızıbekmez, H., Celep, E., Masullo, M., Bassarello, C., Yeşilada, E., and Piacente, S. (2009). Phenylethyl glycosides from *Digitalis lanata*. *Helv. Chim. Acta* 92, 1845–1852. doi: 10.1002/hlca.200900049.

Matsumoto, M., Koga, S., Shoyama, Y., and Nishioka, I. (1987). Phenolic glycoside composition of leaves and callus cultures of *Digitalis purpurea*. *Phytochemistry* 26, 3225–3227.

Meng, Y., Krzysiak, A. J., Durako, M. J., Kunzelman, J. I., and Wright, J. L. C. (2008). Flavones and flavone glycosides from *Halophila johnsonii*. *Phytochemistry* 69, 2603–2608. doi: 10.1016/j.phytochem.2008.07.007.

Morita, J., and Satoh, D. (1968). Studies on *Digitalis* Glycosides. XXVI. Gitoxin Acetates.(1). Deacetylation of Pentaacetylgitoxin. *Chem. Pharm. Bull.* 16, 1056–1061. doi: 10.1248/cpb.16.1056.

Munkert, J., Santiago Franco, M., Nolte, E., Thaís Silva, I., Oliveira Castilho, R., Melo Ottoni, F., et al. (2017). Production of the Cytotoxic Cardenolide Glucoevatromonoside by Semisynthesis and Biotransformation of Evatromonoside by a *Digitalis lanata* Cell Culture. *Planta Med.* 83, 1035–1043. doi: 10.1055/s-0043-109557.

Murai, Y., Kanemoto, T., and Iwashina, T. (2008). Flavone glucuronides from *Plantago hakusanensis* endemic to Japan. *Biochem. Syst. Ecol.* 36, 815–816. doi: 10.1016/j.bse.2008.08.001.

Novković, V. V., Stanojević, L. P., Cakić, M. D., Veljković, V. B., and Stanković, M. Z. (2014). Separation of digoxin by luiquid-luiquid extraction from extracts of foxglove secondary glycosides. *Hem. Ind.* 68, 161–170. doi: 10.2298/HEMIND130422040N.

Oh, J. W., Lee, J. Y., Han, S. H., Moon, Y. H., Kim, Y. G., Woo, E.-R., et al. (2005). Effects of phenylethanoid glycosides from *Digitalis purpurea* L. on the expression of inducible nitric oxide synthase†. *J. Pharm. Pharmacol.* 57, 903–910. doi: 10.1211/0022357056451.

Olvera-García, V., Martín del Campo, S. T., Gutiérrez-Uribe, J. A., and Cardador-Martínez, A. (2015). GC–MS and HPLC–MS-TOF characterization of *Agave atrovirens* extracts. A preliminary study. *Ind. Crop Prod.* 78, 39–47. doi: 10.1016/j.indcrop.2015.09.068.

Pang, X., Cong, Y., Yu, H.-S., Kang, L.-P., Feng, B., Han, B.-X., et al. (2012). Spirostanol saponins derivated from the seeds of *Trigonella foenum-graecum* by β-glucosidase hydrolysis and their inhibitory effects on rat platelet aggregation. *Planta Med.* 78, 276–285. doi: 10.1055/s-0031-1280373.

Perrone, A., Capasso, A., Festa, M., Kemertelidze, E., Pizza, C., Skhirtladze, A., et al. (2012). Antiproliferative steroidal glycosides from *Digitalis ciliata*. *Fitoterapia* 83, 554–562. doi: 10.1016/j.fitote.2011.12.020.

Pommerrenig, B., Eggert, K., and Bienert, G. P. (2019). Boron deficiency effects on sugar, ionome, and phytohormone profiles of vascular and non-vascular leaf tissues of common plantain (*Plantago major* L.). *Int. J. Mol. Sci.* 20, 3882. doi: 10.3390/ijms20163882.

Ravi, B. G., Guardian, M. G. E., Dickman, R., and Wang, Z. Q. (2020). Profiling and structural analysis of cardenolides in two species of *Digitalis* using liquid chromatography coupled with high-resolution mass spectrometry. *J. Chromatogr. A* 1618, 460903. doi: 10.1016/j.chroma.2020.460903.

Rehecho, S., Hidalgo, O., García-Iñiguez de Cirano, M., Navarro, I., Astiasarán, I., Ansorena, D., et al. (2011). Chemical composition, mineral content and antioxidant activity of *Verbena officinalis* L. *LWT-Food Sci. Technol.* 44, 875–882. doi: 10.1016/j.lwt.2010.11.035.

Rhenius, M., Porzel, A., Diettrich, B., and Luckner, M. (1997). 21′-di-dehydro-deacetyllanatoside C, a biotransformation product of deacetyllanatoside C from senescent shoot cultures of *Digitalis lanata*. *Phytochemistry* 44, 1061–1064. doi: 10.1016/S0031-9422(96)00676-0.

Satoh, D., Ishii, H., and Oyama, Y. (1960). Studies on *Digitalis* Glycosides Structure of Purpnigenin and Purprogenin. *Chem. Pharm. Bull.* 8, 657–658. doi: 10.1248/cpb.8.657.

Skhirtladze, A., Kemertelidze, E., Nebieridze, V., and Ganzera, M. (2016). Phenylethanoid Glycosides from the Roots of *Digitalis ciliata* Trautv. *Hel. Chim. Acta* 99, 241–245. doi: 10.1002/hlca.201500288.

Skhirtladze, A. V., Kopaliani, T. A., Nebieridze, V. G., Kemertelidze, E. P., and Ganzera, M. (2017). New steroidal glycosides from pericarp of *Digitalis ferruginea*. *Chem. Nat. Compd.* 53, 1083–1087. doi: 10.1007/s10600-017-2206-x.

Smith, T. W., Antman, E. M., Friedman, P. L., Blatt, C. M., and Marsh, J. D. (1984). *Digitalis* glycosides: Mechanisms and manifestations of toxicity. Part I . *Prog. Cardiovasc. Dis.* 26, 413–458. doi: 10.1016/0033-0620(84)90012-4.

Šuković, D., Knežević, B., Gašić, U., Sredojević, M., Ćirić, I., Todić, S., et al. (2020). Phenolic Profiles of Leaves, Grapes and Wine of Grapevine Variety Vranac (*Vitis vinifera* L.) from Montenegro. *Foods* 9, 138. doi: 10.3390/foods9020138.

Tan, L. (1969). An improved method for the gas chromatographic identification of *Digitalis* cardenolides. *J. Chematogr. A* 45, 68–75. doi: 10.1016/S0021-9673(01)86183-5.

Taskova, R. M., Gotfredsen, C. H., and Jensen, S. R. (2005). Chemotaxonomic markers in Digitalideae (Plantaginaceae). *Phytochemistry* 66, 1440–1447. doi: 10.1016/j.phytochem.2005.04.020.

Templeton, J. F., Setiloane, P., Kumar, V. P. S., Yan, Y., Zeglam, T. H., and LaBella, F. S. (1991). Pregnanes that bind to the digitalis receptor: synthesis of 14-hydroxy-5.beta.,14.beta.-pregnane glycosides from digitoxin and digitoxigenin. *J. Med. Chem.* 34, 2778–2782. doi: 10.1021/jm00113a015.

Theurer, C., Kreis, W., and Reinhard, E. (1998). Effects of digitoxigenin, digoxigenin, and various cardiac glycosides on cardenolide accumulation in shoot cultures of *Digitalis lanata*. *Planta Med.* 64, 705–710. doi: 10.1055/s-2006-957562.

Wang, J., Jiang, W., Liu, Z., Wang, J., Fu, T., and Wang, Y. (2017a). Analysis and identification of chemical constituents of fenugreek by UPLC-IT-MS n and UPLC-Q-TOF-MS. *Chem. Res. Chin. Univ.* 33, 721–730. doi: 10.1007/s40242-017-7136-4.

Wang, C., Zhang, N., Wang, Z., Qi, Z., Zheng, B., Li, P., et al. (2017b). Rapid characterization of chemical constituents of *Platycodon grandiflorum* and its adulterant Adenophora stricta by UPLC-QTOF-MS/MS. *J. Mass Spectrom.* 52, 643–656. doi: 10.1002/jms.3967.

Wang, L., Gao, G., Bai, Y., Luo, W., Lin, C., and Jia, Q. (2011). Fingerprint quality detection of *Solanum* *nigrum* using high-performance liquid chromatography-evaporative light scattering detection. *Pharm. Biol.* 49, 595–601. doi: 10.3109/13880209.2010.535171.

Wang, Q., Xie, J., Fang, S., Shen, X., Li, Q., Wu, X., et al. (2013). Complementary Application of HSCCC and Semi-preparative HPLC for Rapid Separation of Phenylethanoid Glycosides from *Penstemon* *digitalis*. *Chinese Herbal Medicines* 5, 280–285. doi: 10.1016/S1674-6384(13)60041-6.

Wang, Y., Fan, Q., Xiang, J., Huang, H., Chen, S., Liu, B., et al. (2020). Structural characterization and discrimination of *Paris polyphylla* var*. yunnanensis* by a molecular networking strategy coupled with ultra-high-performance liquid chromatography with quadrupole time-of-flight mass spectrometry. *Rapid Commun. Mass Spectrom.* 34, e8760. doi: 10.1002/rcm.8760.

Wang, Y., Liu, Q., Fan, S., Yang, X., Ming, L., Wang, H., et al. (2019). Rapid analysis and characterization of multiple constituents of corn silk aqueous extract using ultra-high-performance liquid chromatography combined with quadrupole time-of-flight mass spectrometry. *J. Sep. Sci.* 42, 3054–3066. doi: 10.1002/jssc.201900407.

Weiler, E. W., and Zenk, M. H. (1976). Radioimmunoassay for the determination of digoxin and related compounds in *Digitalis lanata*. *Phytochemistry* 15, 1537–1545. doi: 10.1016/S0031-9422(00)88933-5.

Xu, R., Yang, Y., Zhang, Y., Ren, F., Xu, J., Yu, N., et al. (2015). New Pregnane Glycosides from *Gymnema sylvestre*. *Molecules* 20, 3050–3066. doi: 10.3390/molecules20023050.

Zengin, G., Cvetanović, A., Gašić, U., Dragićević, M., Stupar, A., Uysal, A., et al. (2020). UHPLC-LTQ OrbiTrap MS analysis and biological properties of *Origanum vulgare* subsp. *viridulum* obtained by different extraction methods. *Ind. Crop Prod.* 154, 112747. doi: 10.1016/j.indcrop.2020.112747.

Zhang, X., Liao, M., Cheng, X., Liang, C., Diao, X., and Zhang, L. (2018). Ultrahigh-performance liquid chromatography coupled with triple quadrupole and time-of-flight mass spectrometry for the screening and identification of the main flavonoids and their metabolites in rats after oral administration of *Cirsium japonicum* DC. extract. *Rapid Commun. Mass Spectrom.* 32, 1451–1461. doi: 10.1002/rcm.8161.

Zhou, J., Yi, H., Zhao, Z.-X., Shang, X.-Y., Zhu, M.-J., Kuang, G.-J., et al. (2018). Simultaneous qualitative and quantitative evaluation of *Ilex kudingcha* C. J. tseng by using UPLC and UHPLC-qTOF-MS/MS. *J. Pharmaceut. Biomed.* 155, 15–26. doi: 10.1016/j.jpba.2018.02.037.
